# Supplementary material for: Distributed harmonic patterns of structure-function dependence orchestrate human consciousness
Source: Commun Biol. 2023 Jan 28;6:117. doi: 10.1038/s42003-023-04474-1 (PMC9884288; doi:10.1038/s42003-023-04474-1)
Supplement: Supplementary file 1 — Supplementary Information [file 42003_2023_4474_MOESM1_ESM.pdf]

## Supplementary Information for

# *Distributed harmonic patterns of structure-function dependence orchestrate human consciousness*

Andrea I. Luppi, Jakub Vohryzek, Morten L. Kringelbach, Pedro A.M. Mediano, Michael M. Craig, Ram Adapa, Robin L. Carhart-Harris, Leor Roseman, Ioannis Pappas, Alexander R.D. Peattie, Anne E. Manktelow, Barbara J. Sahakian, Paola Finoia, Guy B. Williams, Judith Allanson, John D. Pickard, David K. Menon, Selen Atasoy, & Emmanuel A. Stamatakis

Corresponding author: Andrea I. Luppi
Email: al857@cam.ac.uk

# Supplementary Figures

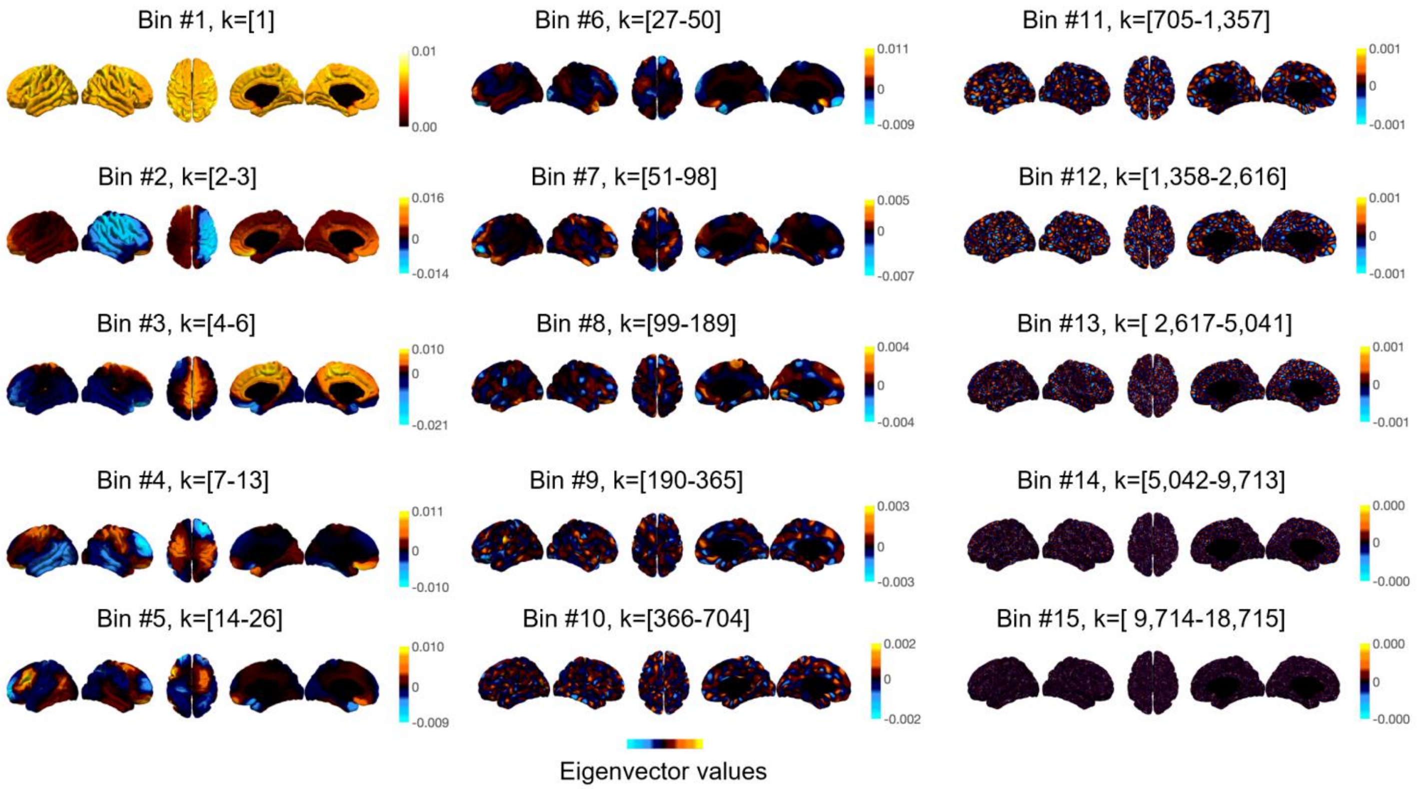


**Supplementary Figure 1**. **Binned connectome harmonics**. Surface projections of connectome harmonics averaged over each of 15 logarithmically spaced bins (with corresponding wavenumbers *k* indicated in braces), showing the progressive increase in complexity and granularity of the connectome harmonic patterns, with increasing spatial frequency.

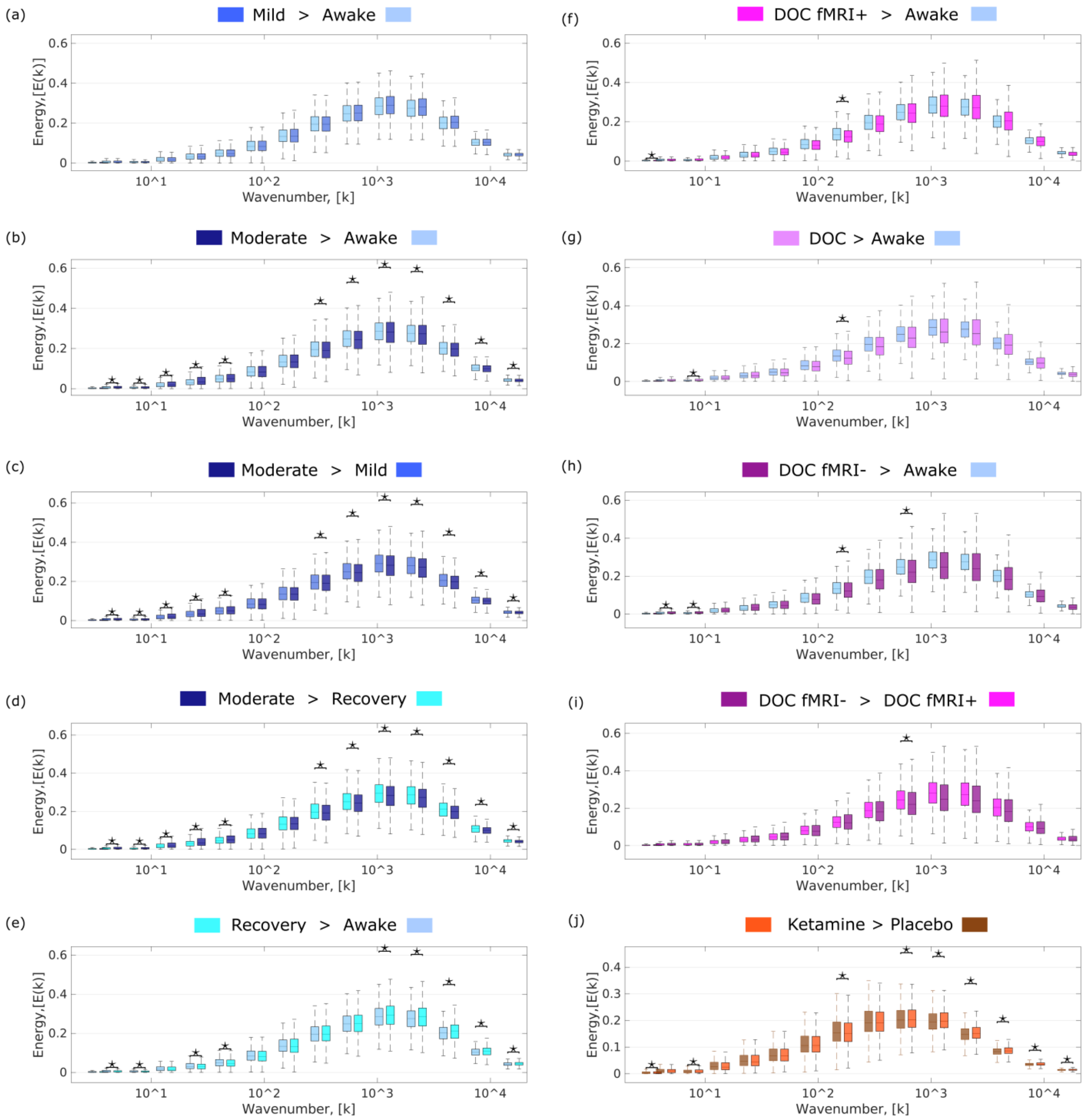


**Supplementary Figure 2**. **Distribution of frequency-specific energy levels across states of consciousness**. (a) Mild propofol sedation (n=15 subjects with 145 timepoints each) vs wakefulness (n=15 subjects with 145 timepoints each). (b) Moderate anaesthesia (n=15 subjects with 145 timepoints each) vs wakefulness (n=15 subjects with 145 timepoints each). (c) Moderate anaesthesia (n=15 subjects with 145 timepoints each) vs mild sedation (n=15 subjects with 145 timepoints each). (d) Moderate anaesthesia (n=15 subjects with 145 timepoints each) vs post-anaesthetic recovery (n=15 subjects with 145 timepoints each). (e) Recovery (n=15 subjects with 145 timepoints each) vs wakefulness (n=15 subjects with 145 timepoints each). (f) DOC patients (n=22 subjects with 295 timepoints each) vs awake healthy controls (n=15 subjects with 145 timepoints each). (g) DOC fMRI+ patients (n=8 subjects with 295 timepoints each) vs awake healthy controls (n=15 subjects with 145 timepoints each). (h) DOC fMRI- patients (n=14 subjects with 295 timepoints each) vs awake healthy controls (n=15 subjects with 145 timepoints each). (i) fMRI- DOC patients (n=14 subjects with 295 timepoints each) vs fMRI+ DOC patients (n=8 subjects with 295 timepoints each). (j) Ketamine (n=20 subjects with 295 timepoints each) vs placebo (n=20 subjects with 295 timepoints each). * $p < 0.05$, FDR-corrected across 15 frequency bins. Pairs of conditions (states of consciousness) were compared with linear mixed effects modelling, by treating condition as a fixed effect and subjects as random effects. Timepoints were also included as random effects, nested within subjects. Boxplots display data distributions across subjects and timepoints, for each condition: central line indicates the median; box edges indicate the 25th and 75th percentiles; whiskers: 1.5x inter-quartile interval.

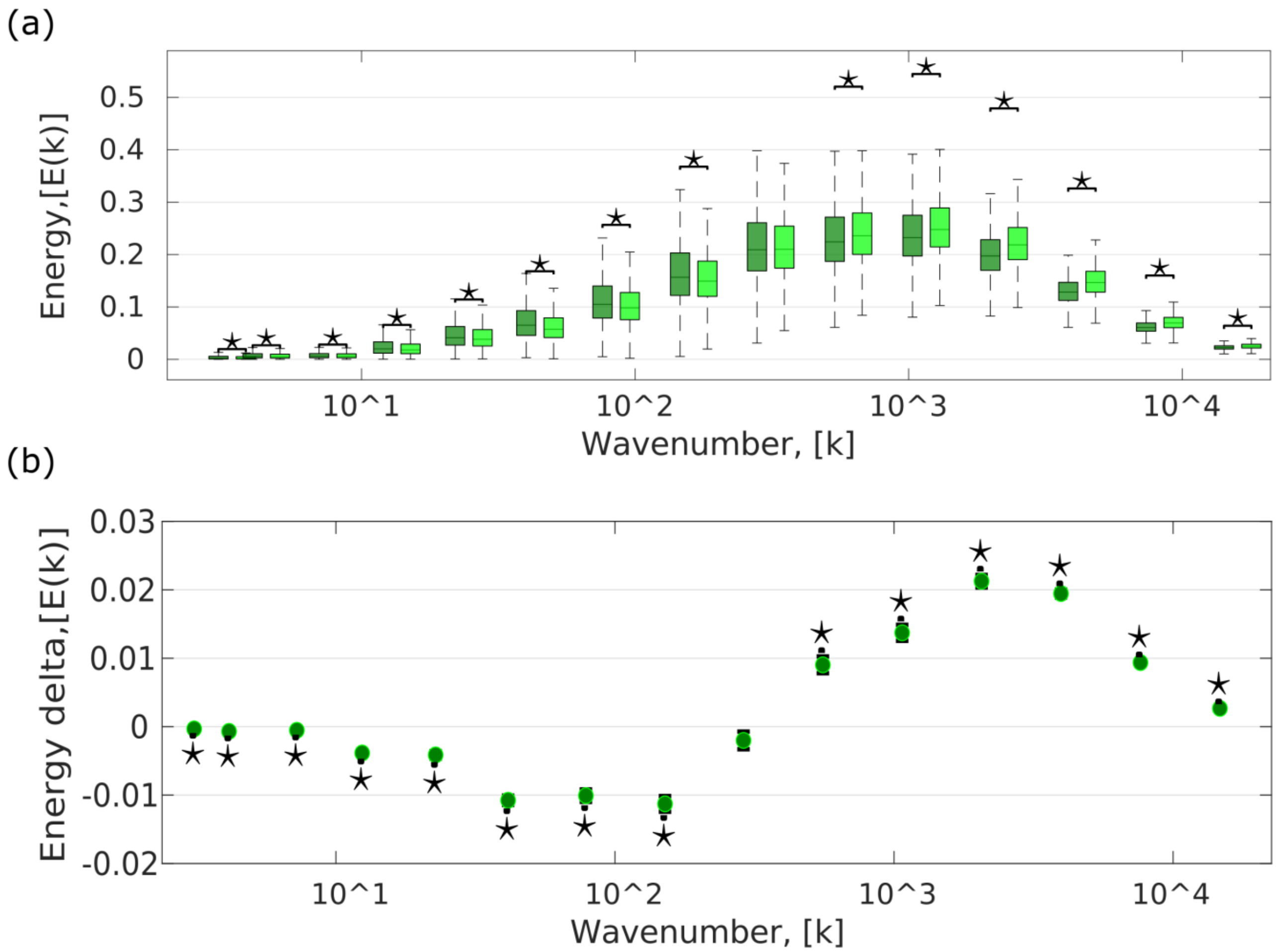


**Supplementary Figure 3**. **Connectome harmonic energy signature of LSD.** Re-derived from the same data used by Atasoy and colleagues (2017) [1]. (a) Boxplots display data distributions across subjects and timepoints, for each condition (n=15 subjects with 435 timepoints each, for both LSD and placebo): central line indicates the median; box edges indicate the 25th and 75th percentiles; whiskers: 1.5x inter-quartile interval. (b) Statistical estimates from linear mixed effects modelling between pairs of conditions (states of consciousness), treating condition as a fixed effect and subjects as random effects. Timepoints were also included as random effects, nested within subjects. Error bars represent 95% confidence interval from the LME model. * $p < 0.05$, FDR-corrected across 15 frequency bins.

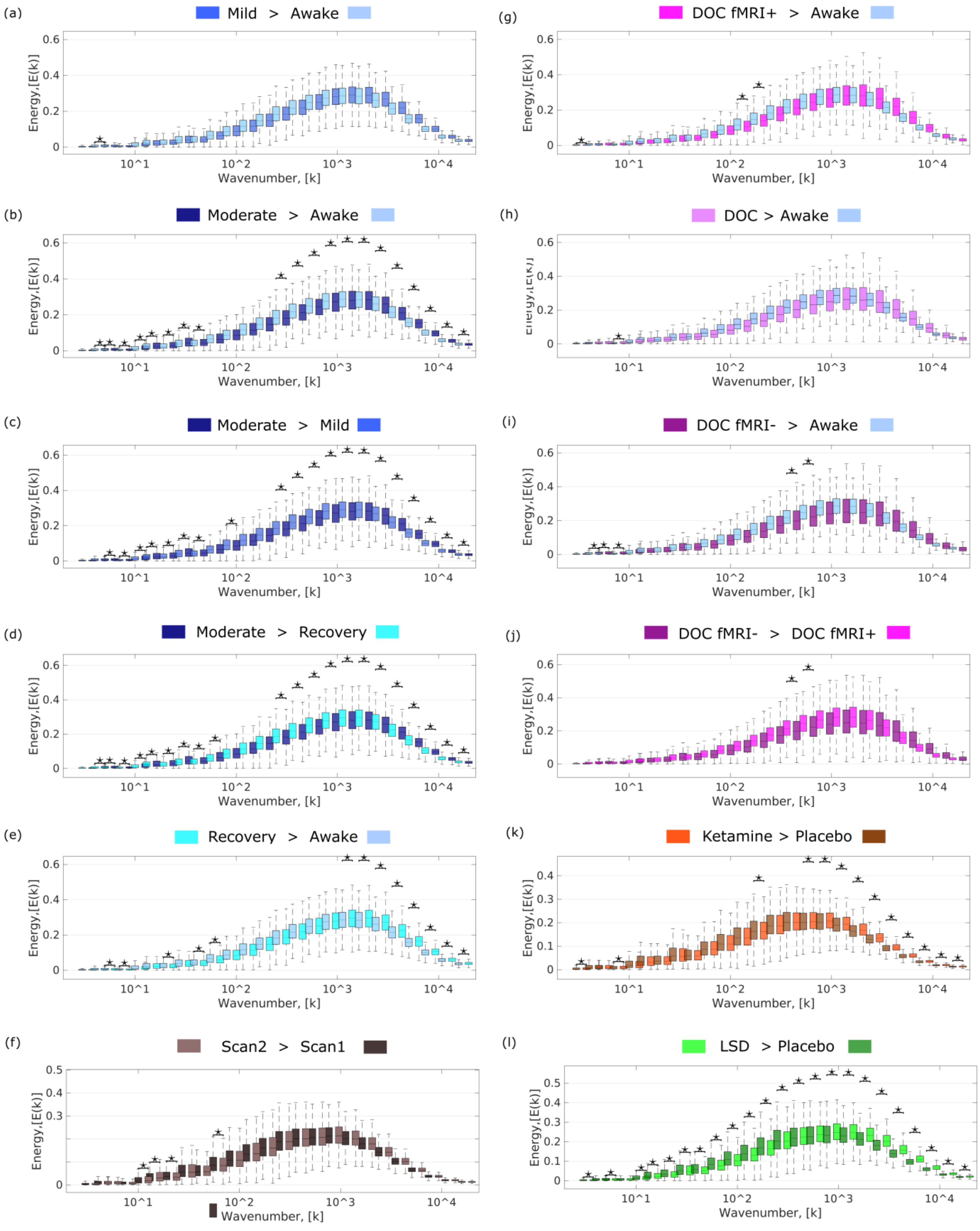
(a)
Mild > Awake
(b)
Moderate > Awake
(c)
Moderate > Mild
(d)
Moderate > Recovery
(e)
Recovery > Awake
(f)
Scan2 > Scan1
(g)
DOC fMRI+ > Awake
(h)
DOC > Awake
(i)
DOC fMRI- > Awake
(j)
DOC fMRI- > DOC fMRI+
(k)
Ketamine > Placebo
(l)
LSD > Placebo
Energy,[E(k)]
Wavenumber, [k]
10^1
10^2
10^3
10^4

**Supplementary Figure 4**. **Distribution of frequency-specific connectome harmonic energy across states of consciousness when using 25 bins.** (a) Mild propofol sedation (n=15 subjects with 145 timepoints each) vs wakefulness (n=15 subjects with 145 timepoints each). (b) Moderate anaesthesia (n=15 subjects with 145 timepoints each) vs wakefulness (n=15 subjects with 145 timepoints each). (c) Moderate anaesthesia (n=15 subjects with 145 timepoints each) vs mild sedation (n=15 subjects with 145 timepoints each). (d) Moderate anaesthesia (n=15 subjects with 145 timepoints each) vs post-anaesthetic recovery (n=15 subjects with 145 timepoints each). (e) Recovery (n=15 subjects with 145 timepoints each) vs wakefulness (n=15 subjects with 145 timepoints each). (f) Test-retest scan 2 (n=18 subjects with 155 timepoints each) vs scan 1 (n=18 subjects with 155 timepoints each); (g) DOC patients (n=22 subjects with 295 timepoints each) vs awake healthy controls (n=15 subjects with 145 timepoints each). (h) DOC fMRI+ patients (n=8 subjects with 295 timepoints each) vs awake healthy controls (n=15 subjects with 145 timepoints each). (i) DOC fMRI- patients (n=14 subjects with 295 timepoints each) vs awake healthy controls (n=15 subjects with 145 timepoints each). (j) fMRI- DOC patients (n=14 subjects with 295 timepoints each) vs fMRI+ DOC patients (n=8 subjects with 295 timepoints each). (k) Ketamine (n=20 subjects with 295 timepoints each) vs placebo (n=20 subjects with 295 timepoints each). (l) LSD (n=15 subjects with 435 timepoints each) vs placebo (n=15 subjects with 435 timepoints each). * $p < 0.05$, FDR-corrected across 25 logarithmically spaced frequency bins. Pairs of conditions (states of consciousness) were compared with linear mixed effects modelling, by treating condition as a fixed effect and subjects as random effects. Timepoints were also included as random effects, nested within subjects. Boxplots display data distributions across subjects and timepoints, for each condition: central line indicates the median; box edges indicate the 25th and 75th percentiles; whiskers: 1.5x inter-quartile interval.

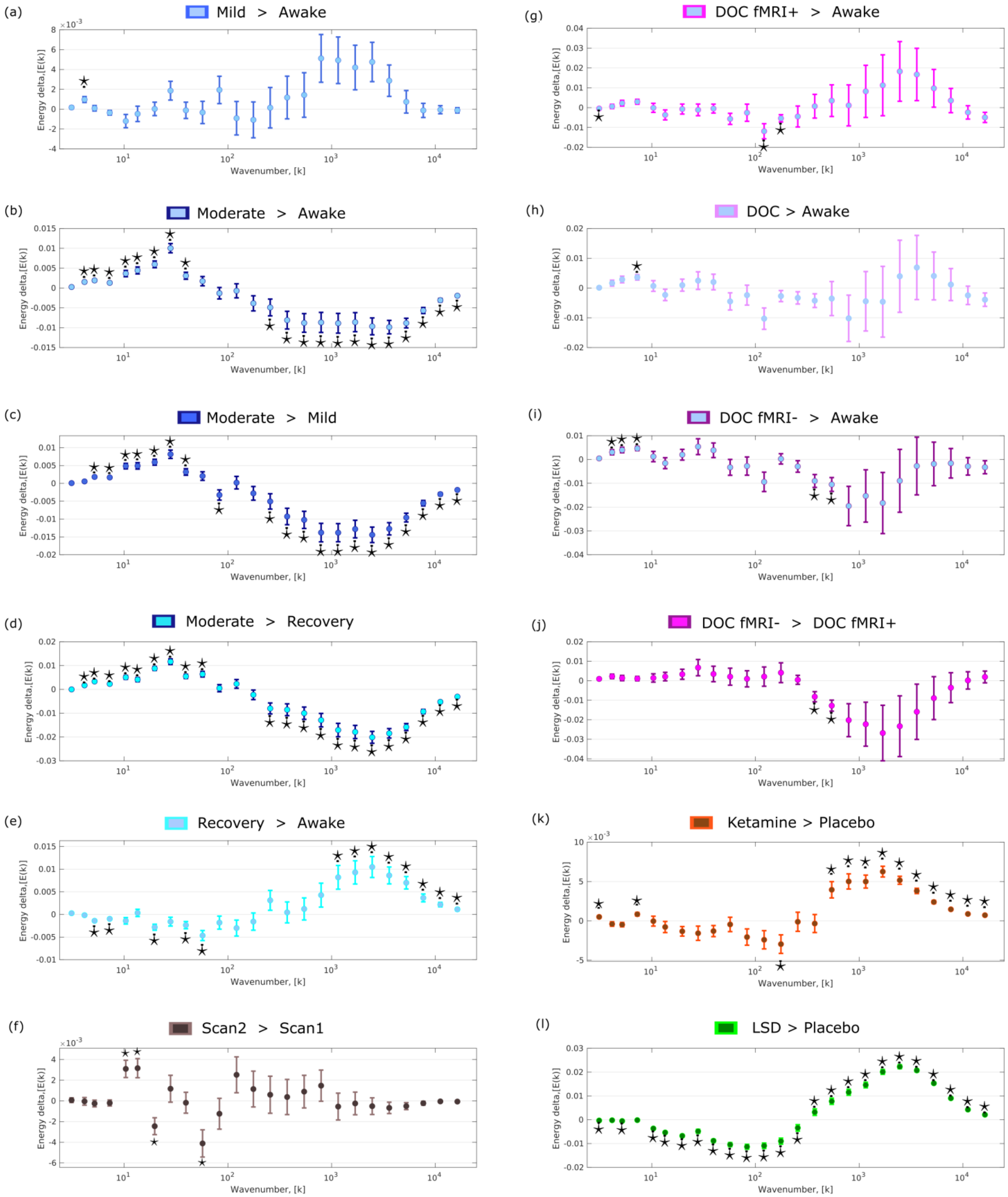
(a)
Mild > Awake
(b)
Moderate > Awake
(c)
Moderate > Mild
(d)
Moderate > Recovery
(e)
Recovery > Awake
(f)
Scan2 > Scan1
(g)
DOC fMRI+ > Awake
(h)
DOC > Awake
(i)
DOC fMRI- > Awake
(j)
DOC fMRI- > DOC fMRI+
(k)
Ketamine > Placebo
(l)
LSD > Placebo
Energy delta,[E(k)]
Wavenumber, [k]

**Supplementary Figure 5**. **Replication of frequency-specific changes of connectome harmonic energy across states of consciousness when using 25 bins.** Plots show the statistical estimates (fixed effect of condition) and 95% CIs from linear mixed effects modelling between pairs of conditions (states of consciousness), treating condition as a fixed effect and subjects as random effects. Timepoints were also included as random effects, nested within subjects. (a) Mild propofol sedation (n=15 subjects with 145 timepoints each) > wakefulness (n=15 subjects with 145 timepoints each). (b) Moderate anaesthesia (n=15 subjects with 145 timepoints each) > wakefulness (n=15 subjects with 145 timepoints each). (c) Moderate anaesthesia (n=15 subjects with 145 timepoints each) > mild sedation (n=15 subjects with 145 timepoints each). (d) Moderate anaesthesia (n=15 subjects with 145 timepoints each) > post-anaesthetic recovery (n=15 subjects with 145 timepoints each). (e) Recovery (n=15 subjects with 145 timepoints each) > wakefulness (n=15 subjects with 145 timepoints each). (f) Test-retest scan 2 (n=18 subjects with 155 timepoints each) > scan 1 (n=18 subjects with 155 timepoints each); (g) DOC patients (n=22 subjects with 295 timepoints each) > awake healthy controls (n=15 subjects with 145 timepoints each). (h) DOC fMRI+ patients (n=8 subjects with 295 timepoints each) > awake healthy controls (n=15 subjects with 145 timepoints each). (i) DOC fMRI- patients (n=14 subjects with 295 timepoints each) > awake healthy controls (n=15 subjects with 145 timepoints each). (j) fMRI- DOC patients (n=14 subjects with 295 timepoints each) > fMRI+ DOC patients (n=8 subjects with 295 timepoints each). (k) Ketamine (n=20 subjects with 295 timepoints each) > placebo (n=20 subjects with 295 timepoints each). (l) LSD (n=15 subjects with 435 timepoints each) vs placebo (n=15 subjects with 435 timepoints each). * $p < 0.05$, FDR-corrected across 25 logarithmically spaced frequency bins.

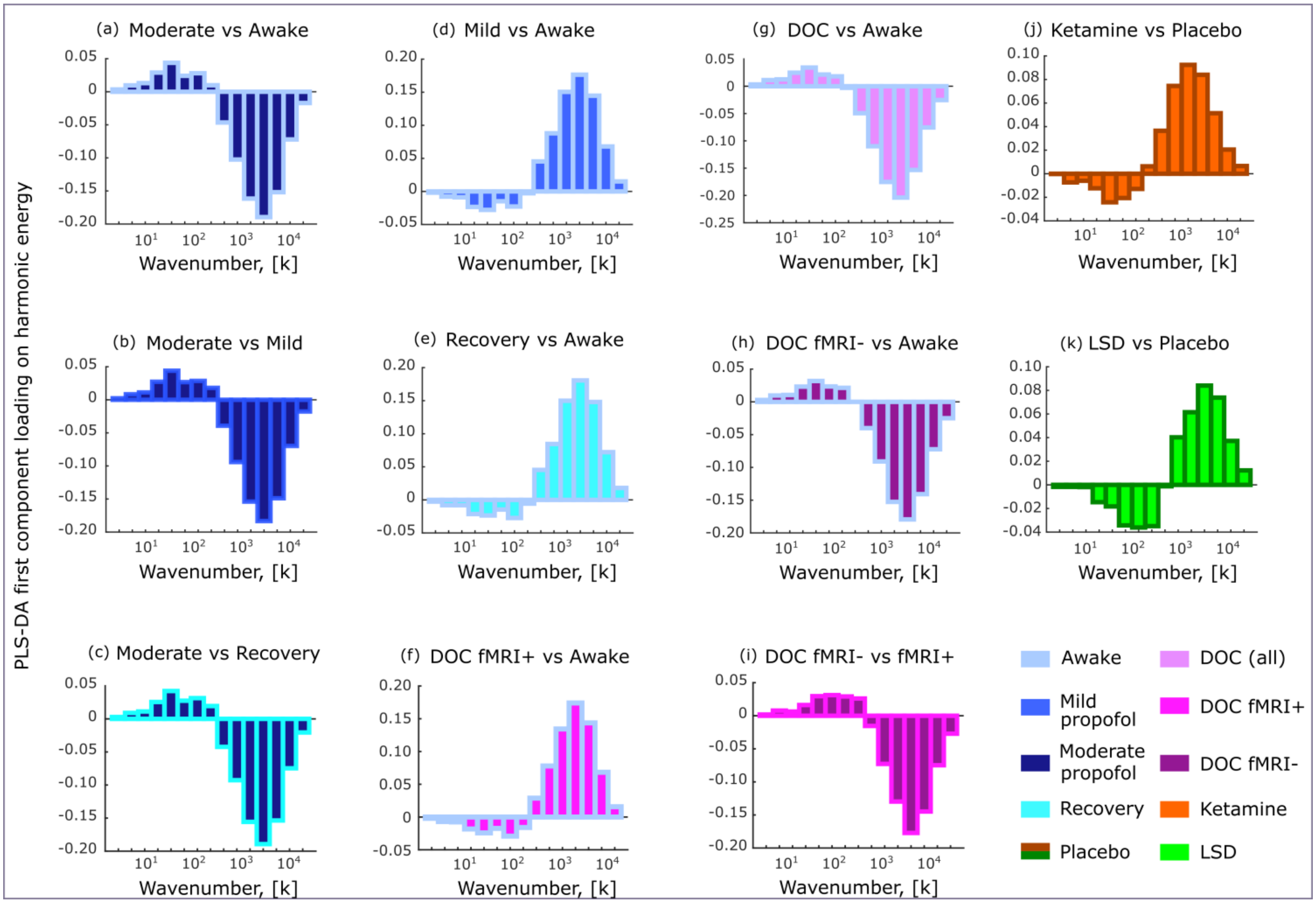


**Supplementary Figure 6. Similar and opposite multivariate signatures of connectome harmonic energy for different states of consciousness.** (a) Moderate anaesthesia > wakefulness. (b) Moderate anaesthesia > mild sedation. (c) Moderate anaesthesia > post-anaesthetic recovery. (d) Mild sedation > wakefulness. (e) Post-anaesthetic recovery > wakefulness. (f) DOC fMRI+ patients > awake healthy controls. (g) DOC patients > awake healthy controls. (h) DOC fMRI- patients > awake healthy controls. (i) fMRI- > fMRI+ DOC patients. (j) Ketamine > placebo. (k) LSD > placebo. Bar colour indicates the target state; contours indicate the reference state.

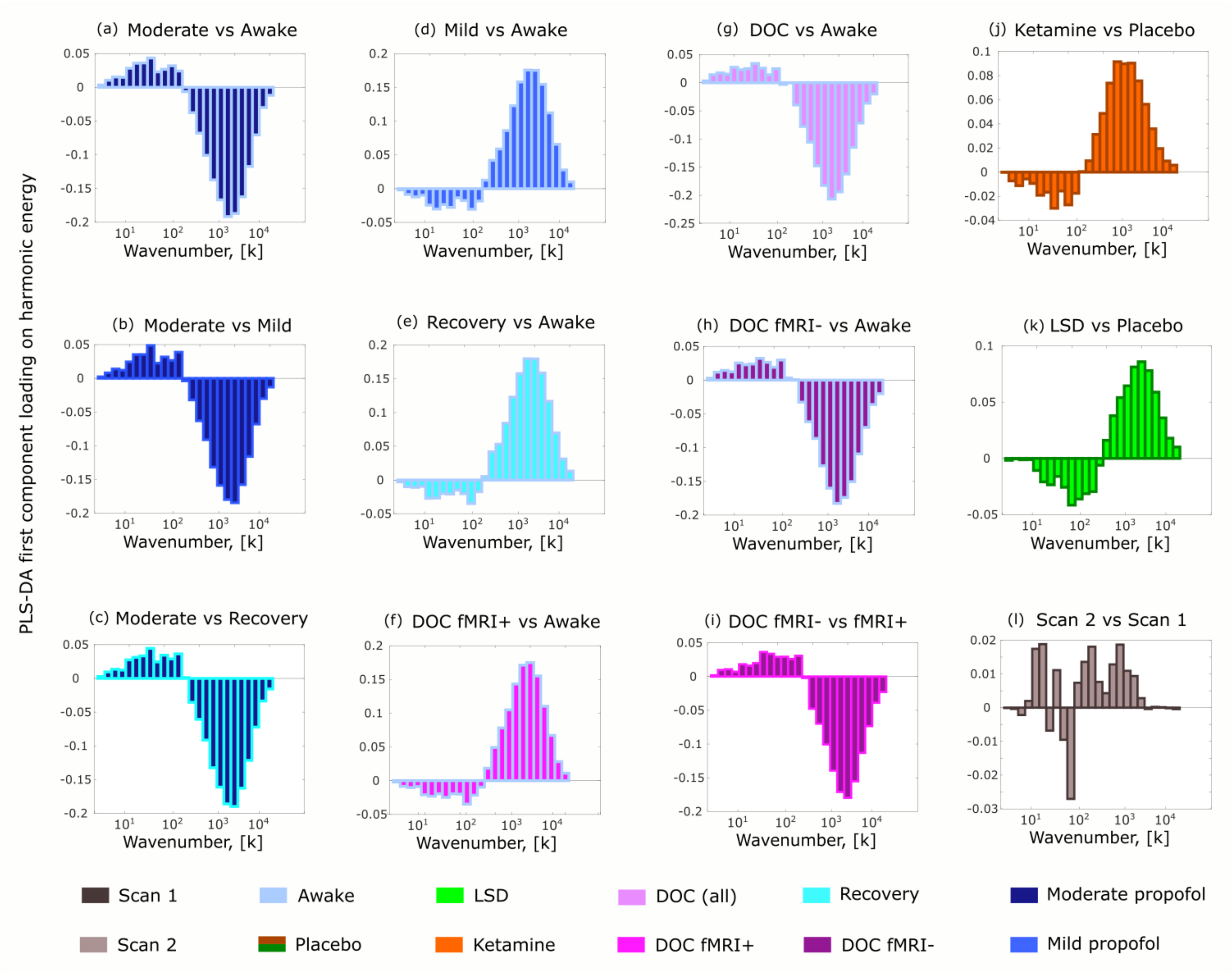


**Supplementary Figure 7. Replication of multivariate signatures of connectome harmonic energy for different states of consciousness, when using 25 bins.** (a) Moderate anaesthesia > wakefulness. (b) Moderate anaesthesia > mild sedation. (c) Moderate anaesthesia > post-anaesthetic recovery. (d) Mild sedation > wakefulness. (e) Post-anaesthetic recovery > wakefulness. (f) DOC fMRI+ patients > awake healthy controls. (g) DOC patients > awake healthy controls. (h) DOC fMRI- patients > awake healthy controls. (i) fMRI- > fMRI+ DOC patients. (j) Ketamine > placebo. (k) LSD > placebo. (l) Test-retest scan2 > scan 1. Bar colour indicates the target state; contours indicate the reference state.

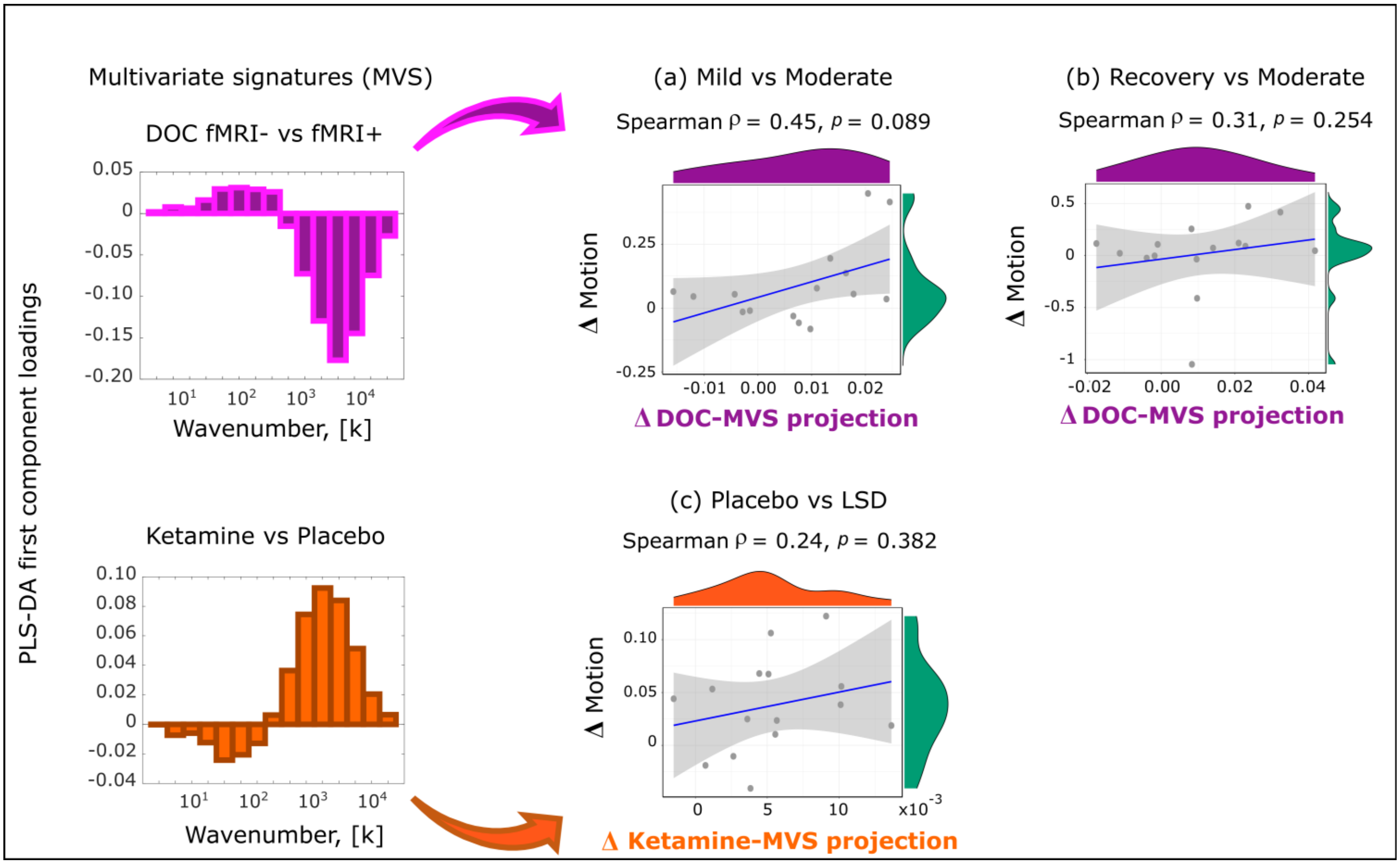


**Supplementary Figure 8. Change in projection onto cross-dataset multivariate energy signature (MVS) does not significantly correlate with differences in subject motion in the scanner.** (a) Scatterplot of delta in connectome harmonic energy projection onto the MVS derived from the DOC dataset, versus the delta in head motion (moderate anaesthesia minus mild anaesthesia; n = 15 subjects). (b) Scatterplot of delta in connectome harmonic energy projection onto the MVS derived from the DOC dataset, versus the delta in head motion (moderate anaesthesia minus recovery; n=15 subjects). (c) Scatterplot of delta in connectome harmonic energy projection onto the MVS derived from the ketamine dataset, versus the delta in head motion (LSD minus placebo; n=15 subjects). Shading indicates 95% confidence interval.

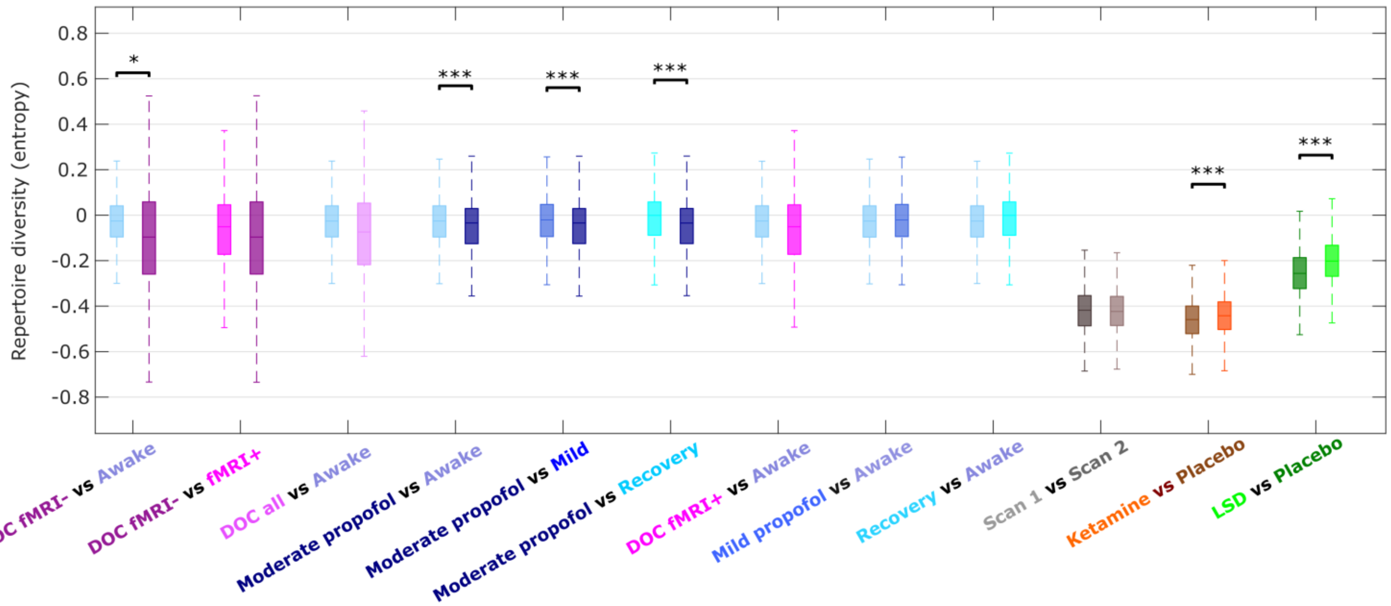


**Supplementary Figure 9. Distribution of repertoire diversity across states of consciousness.** Boxplots display data distributions across subjects and timepoints, for each condition: central line indicates the median; box edges indicate the 25th and 75th percentiles; whiskers: 1.5x inter-quartile interval. *** $p$ < 0.001; ** $p$ < 0.01; * $p$ < 0.05; ˙ $p$ < 0.10. Pairs of conditions (states of consciousness) were compared with linear mixed effects modelling, by treating condition as a fixed effect and subjects as random effects. Timepoints were also included as random effects, nested within subjects. Error bars represent 95% confidence interval from the LME model. Wakefulness, mild sedation, moderate anaesthesia, and recovery: n=15 subjects with 145 timepoints each, for each condition. DOC fMRI+ patients: n=8 subjects with 295 timepoints each; DOC fMRI- patients: n=14 subjects with 295 timepoints each. Test-retest scan 1 and scan 2: n=18 subjects with 155 timepoints each, for each condition. Ketamine and placebo: n=20 subjects with 295 timepoints each, for each condition. LSD and placebo: n=15 subjects with 435 timepoints each, for each condition.

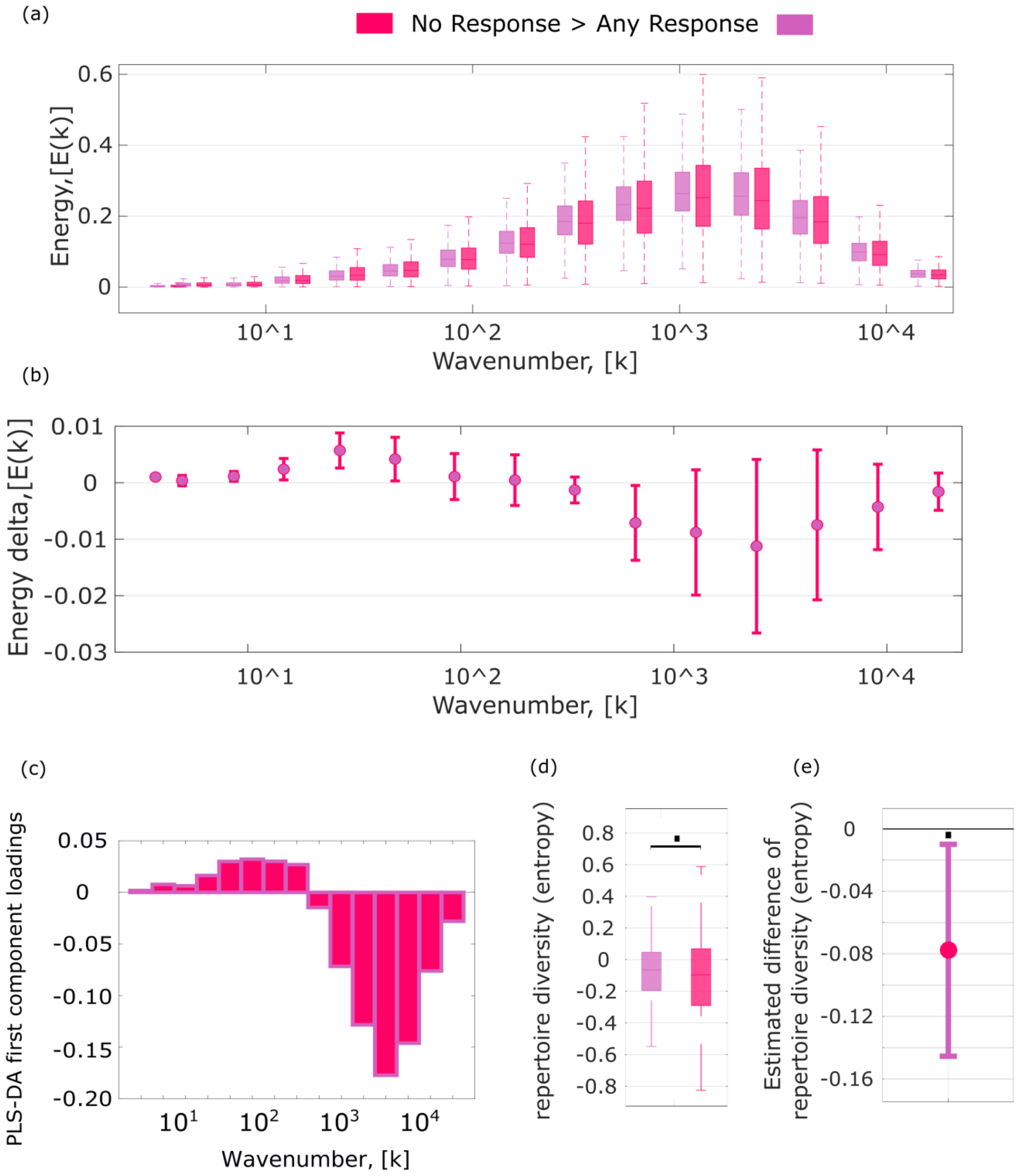


**Supplementary Figure 10**. **Connectome harmonic differences between DOC patients based on combined clinical and fMRI classification.** (a) Distribution of frequency-specific energy of connectome harmonics between patients who are both UWS and fMRI- ("No response", n=8 patients with 295 timepoints each) and all other patients ("Any response", n=14 patients with 295 timepoints each). Boxplots display data distributions across subjects and timepoints, for each condition: central line indicates the median; box edges indicate the 25th and 75th percentiles; whiskers: 1.5x inter-quartile interval. (b) Plots show the statistical estimates (fixed effect of condition) and 95% CIs from linear mixed effects modelling between pairs of conditions (states of consciousness), treating condition as a fixed effect and subjects as random effects. Timepoints were also included as random effects, nested within subjects. (c) Multivariate energy signature (MVS) for best discriminating between the two groups of patients. (d) Boxplots display data distributions across subjects and timepoints, for each condition: central line indicates the median; box edges indicate the 25th and 75th percentiles; whiskers: 1.5x inter-quartile interval. (e) Statistical estimate (fixed effect of condition) and 95% CI from linear mixed effects modelling for the difference in diversity (entropy) of the full connectome harmonic repertoire, which is diminished in fMRI- UWS patients compared with the other DOC patients. · $p < 0.10$.

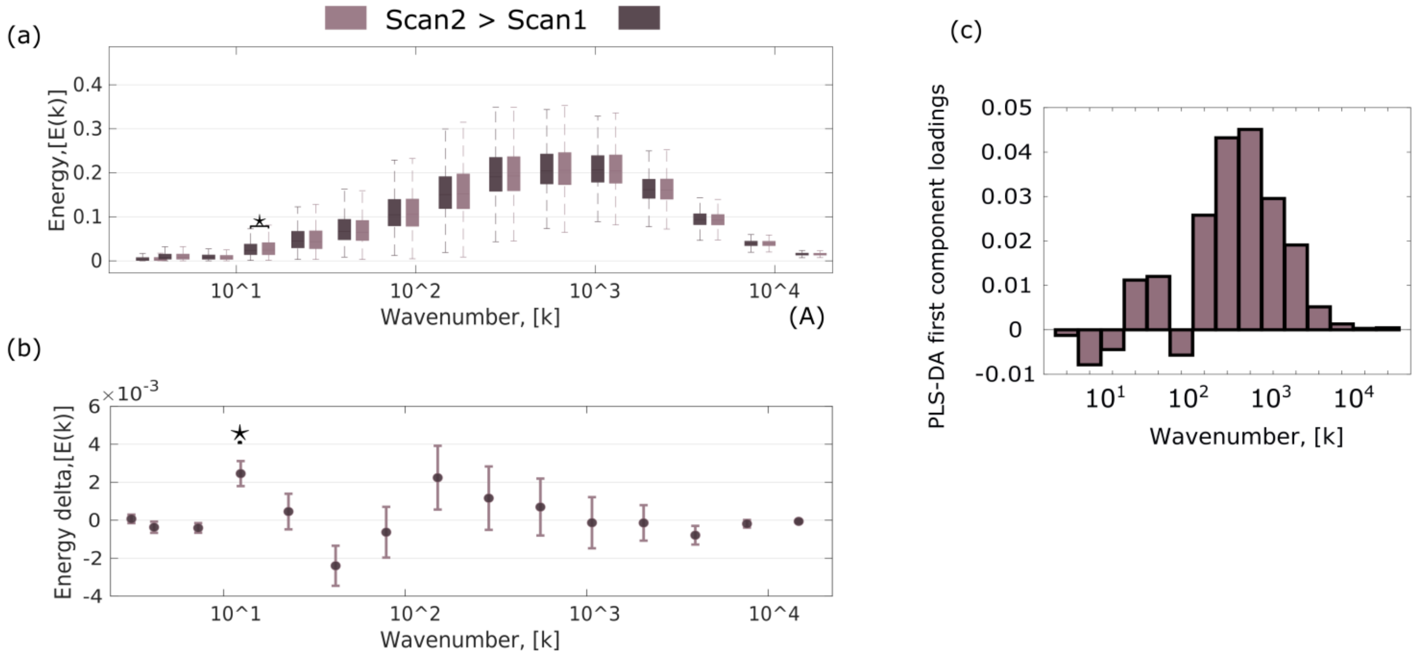


**Supplementary Figure 11**. **Stable energy levels across two scans of the same individuals**. (a) Distributions of frequency-specific energy of connectome harmonics for two different scans of the same individuals (n=18 subjects with 155 timepoints each, for both scan 1 and scan 2). Boxplots display data distributions across subjects and timepoints, for each condition: central line indicates the median; box edges indicate the 25th and 75th percentiles; whiskers: 1.5x inter-quartile interval. (b) Statistical estimates (fixed effect of condition) from linear mixed effects modelling between pairs of conditions (states of consciousness), treating condition as a fixed effect and subjects as random effects. Timepoints were also included as random effects, nested within subjects. Error bars represent 95% confidence interval from the LME model. (c) Multivariate energy signature (MVS) for discriminating between the first and second scan. * $p < 0.05$ (FDR-corrected across 15 frequency bins, for the frequency-specific analysis).

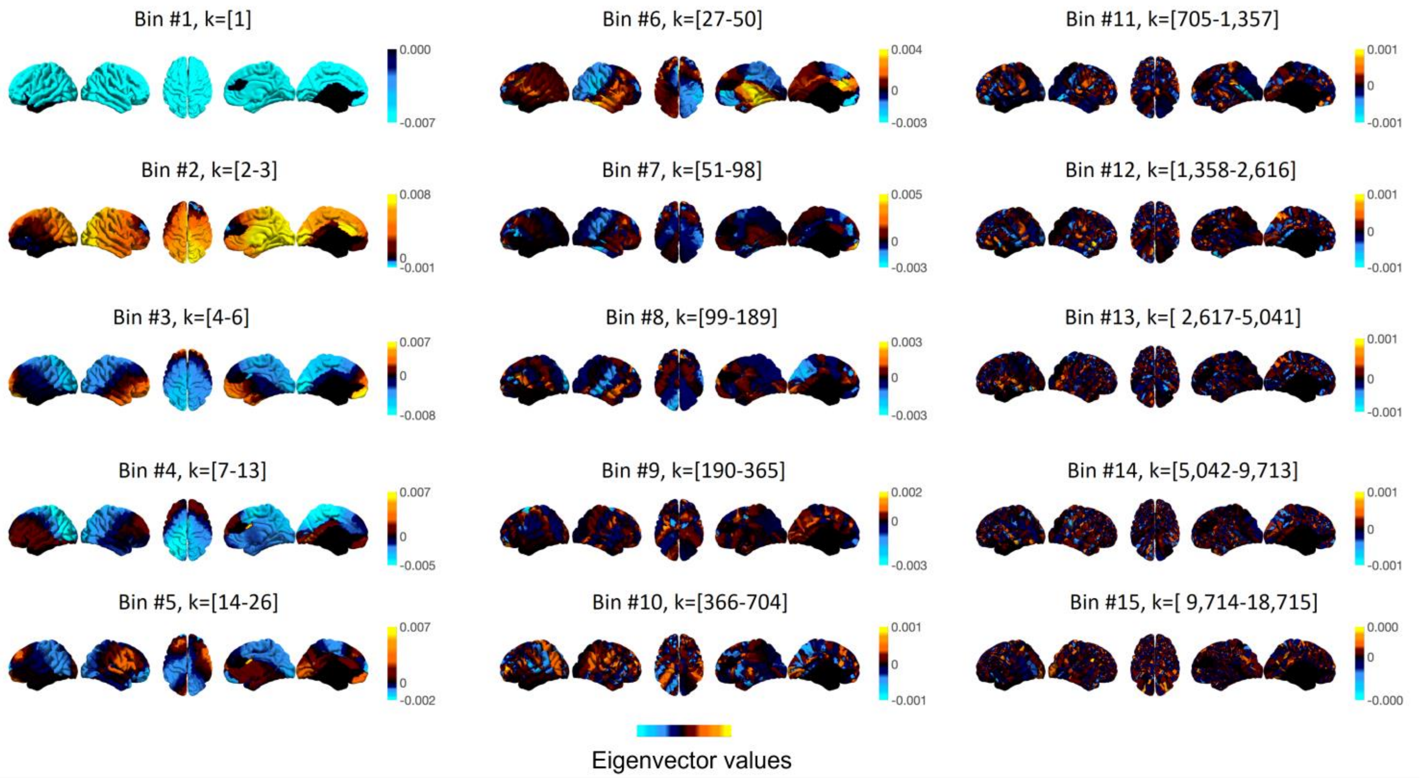


**Supplementary Figure 12**. **Rotated connectome harmonics**. Surface projections of connectome harmonics averaged over each of 15 logarithmically spaced bins (with corresponding wavenumbers *k* indicated in braces), showing the progressive increase in complexity and granularity of the connectome harmonic patterns, with increasing spatial frequency. Each harmonic was projected on a sphere and the sphere was then randomly rotated before back-projecting each harmonic on the brain surface (Methods).

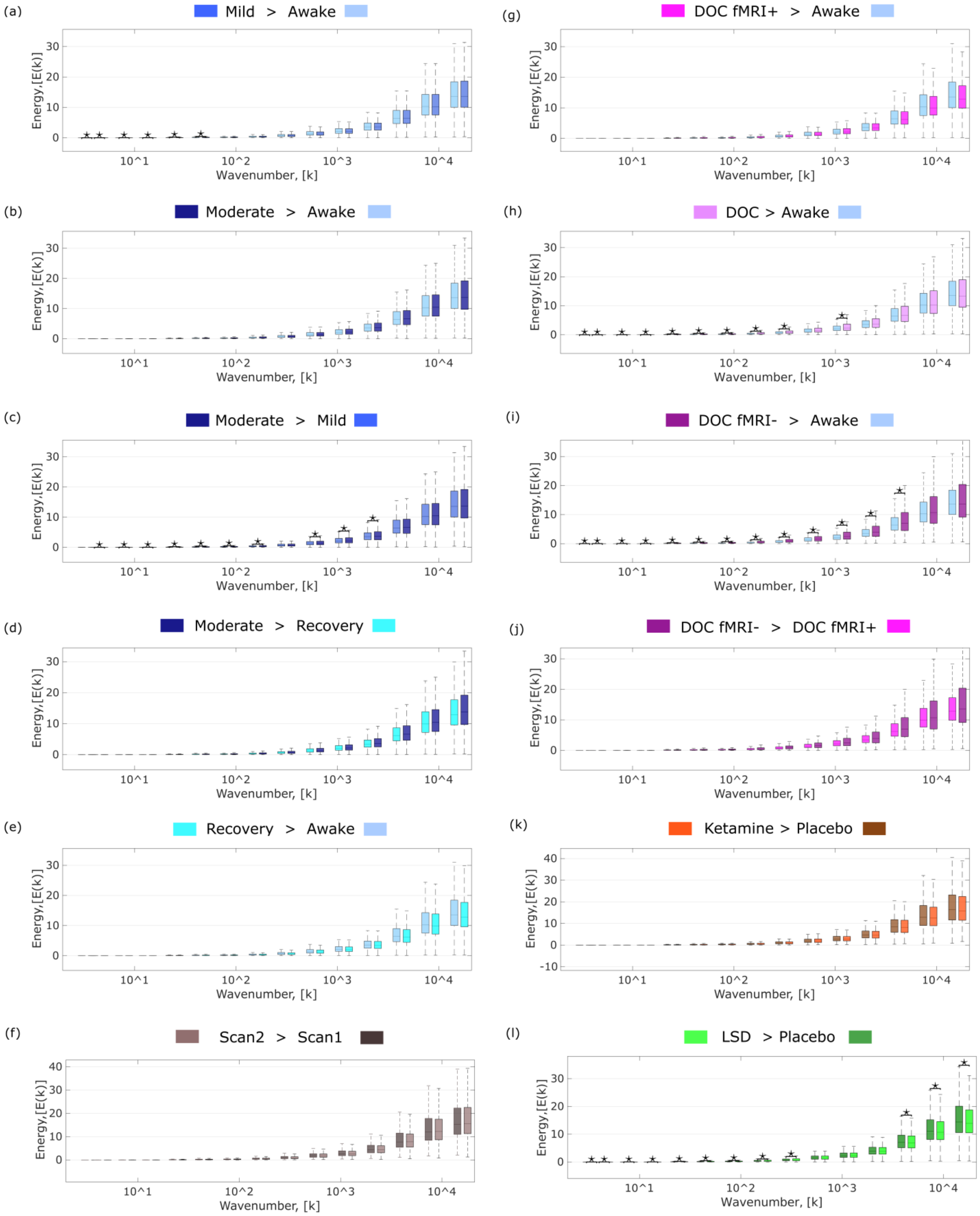
(a)
Mild > Awake
(b)
Moderate > Awake
(c)
Moderate > Mild
(d)
Moderate > Recovery
(e)
Recovery > Awake
(f)
Scan2 > Scan1
(g)
DOC fMRI+ > Awake
(h)
DOC > Awake
(i)
DOC fMRI- > Awake
(j)
DOC fMRI- > DOC fMRI+
(k)
Ketamine > Placebo
(l)
LSD > Placebo
Energy,[E(k)]
Wavenumber, [k]
10^1
10^2
10^3
10^4

**Supplementary Figure 13**. **Distribution of frequency-specific energy changes across states of consciousness, for the rotated harmonics.** ((a) Mild propofol sedation (n=15 subjects with 145 timepoints each) vs wakefulness (n=15 subjects with 145 timepoints each). (b) Moderate anaesthesia (n=15 subjects with 145 timepoints each) vs wakefulness (n=15 subjects with 145 timepoints each). (c) Moderate anaesthesia (n=15 subjects with 145 timepoints each) vs mild sedation (n=15 subjects with 145 timepoints each). (d) Moderate anaesthesia (n=15 subjects with 145 timepoints each) vs post-anaesthetic recovery (n=15 subjects with 145 timepoints each). (e) Recovery (n=15 subjects with 145 timepoints each) vs wakefulness (n=15 subjects with 145 timepoints each). (f) Test-retest scan 2 (n=18 subjects with 155 timepoints each) vs scan 1 (n=18 subjects with 155 timepoints each); (g) DOC patients (n=22 subjects with 295 timepoints each) vs awake healthy controls (n=15 subjects with 145 timepoints each). (h) DOC fMRI+ patients (n=8 subjects with 295 timepoints each) vs awake healthy controls (n=15 subjects with 145 timepoints each). (i) DOC fMRI- patients (n=14 subjects with 295 timepoints each) vs awake healthy controls (n=15 subjects with 145 timepoints each). (j) fMRI- DOC patients (n=14 subjects with 295 timepoints each) vs fMRI+ DOC patients (n=8 subjects with 295 timepoints each). (k) Ketamine (n=20 subjects with 295 timepoints each) vs placebo (n=20 subjects with 295 timepoints each). (l) LSD (n=15 subjects with 435 timepoints each) vs placebo (n=15 subjects with 435 timepoints each). Pairs of conditions (states of consciousness) were compared with linear mixed effects modelling, by treating condition as a fixed effect and subjects as random effects. Timepoints were also included as random effects, nested within subjects. Boxplots display data distributions across subjects and timepoints, for each condition: central line indicates the median; box edges indicate the 25th and 75th percentiles; whiskers: 1.5x inter-quartile interval. * $p < 0.05$, FDR-corrected across 15 frequency bins.

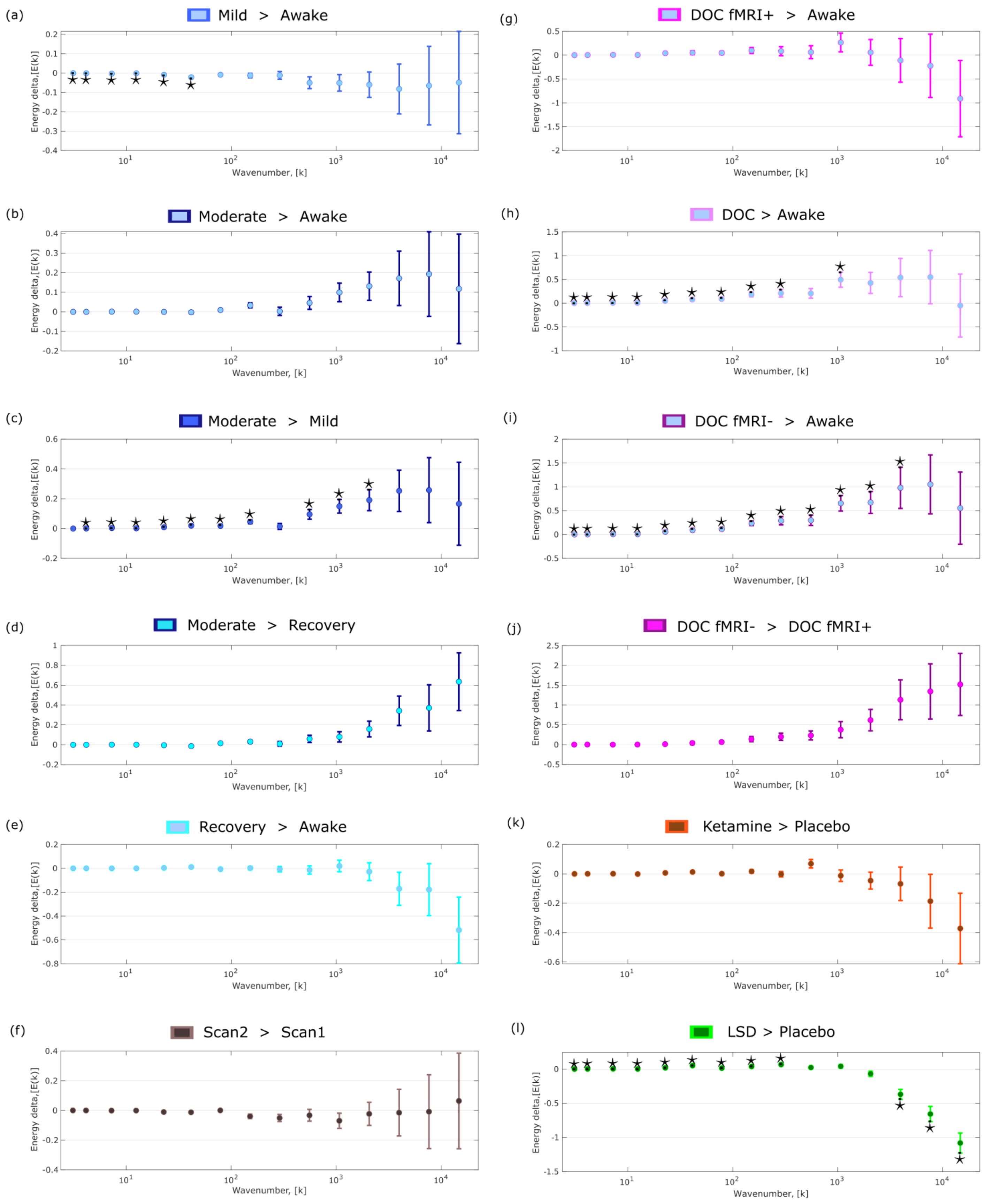

(a)
Mild > Awake
(b)
Moderate > Awake
(c)
Moderate > Mild
(d)
Moderate > Recovery
(e)
Recovery > Awake
(f)
Scan2 > Scan1
(g)
DOC fMRI+ > Awake
(h)
DOC > Awake
(i)
DOC fMRI- > Awake
(j)
DOC fMRI- > DOC fMRI+
(k)
Ketamine > Placebo
(l)
LSD > Placebo
Energy delta,[E(k)]
Wavenumber, [k]

**Supplementary Figure 14**. **No consistent frequency-specific energy changes are observed across states of consciousness, for the rotated harmonics.** Plots show the statistical estimates (fixed effect of condition) and 95% CIs from linear mixed effects modelling between pairs of conditions (states of consciousness), treating condition as a fixed effect and subjects as random effects. Timepoints were also included as random effects, nested within subjects. (a) Mild propofol sedation (n=15 subjects with 145 timepoints each) > wakefulness (n=15 subjects with 145 timepoints each). (b) Moderate anaesthesia (n=15 subjects with 145 timepoints each) > wakefulness (n=15 subjects with 145 timepoints each). (c) Moderate anaesthesia (n=15 subjects with 145 timepoints each) > mild sedation (n=15 subjects with 145 timepoints each). (d) Moderate anaesthesia (n=15 subjects with 145 timepoints each) > post-anaesthetic recovery (n=15 subjects with 145 timepoints each). (e) Recovery (n=15 subjects with 145 timepoints each) > wakefulness (n=15 subjects with 145 timepoints each). (f) Test-retest scan 2 (n=18 subjects with 155 timepoints each) > scan 1 (n=18 subjects with 155 timepoints each); (g) DOC patients (n=22 subjects with 295 timepoints each) > awake healthy controls (n=15 subjects with 145 timepoints each). (h) DOC fMRI+ patients (n=8 subjects with 295 timepoints each) > awake healthy controls (n=15 subjects with 145 timepoints each). (i) DOC fMRI- patients (n=14 subjects with 295 timepoints each) > awake healthy controls (n=15 subjects with 145 timepoints each). (j) fMRI- DOC patients (n=14 subjects with 295 timepoints each) > fMRI+ DOC patients (n=8 subjects with 295 timepoints each). (k) Ketamine (n=20 subjects with 295 timepoints each) > placebo (n=20 subjects with 295 timepoints each). (l) LSD (n=15 subjects with 435 timepoints each) vs placebo (n=15 subjects with 435 timepoints each). * $p < 0.05$, FDR-corrected across 15 logarithmically spaced frequency bins.

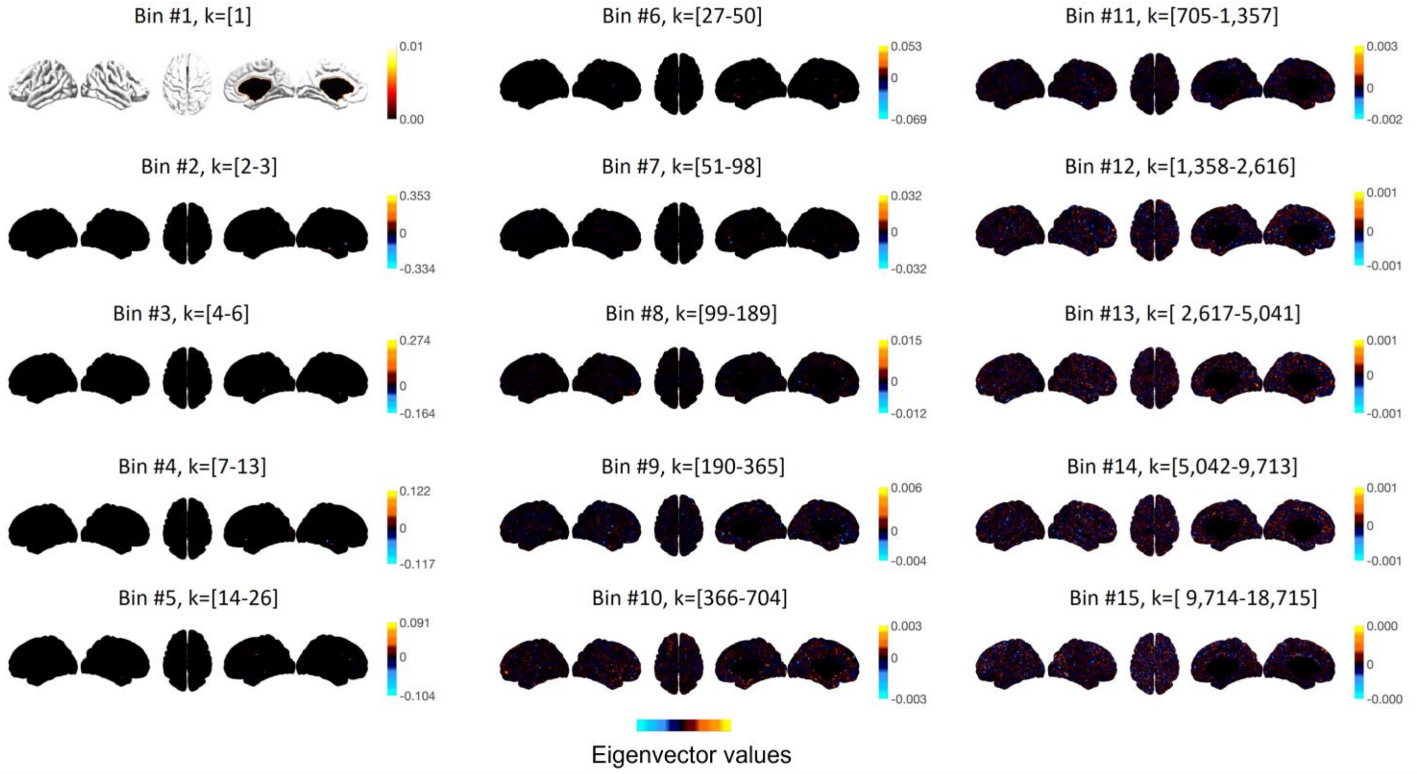


**Supplementary Figure 15**. **Binned connectome harmonics from a randomised connectome**. Surface projections of connectome harmonics averaged over each of 15 logarithmically spaced bins (with corresponding wavenumbers *k* indicated in braces; note that only the first 14 bins are used for analysis). Note how the colour is largely uniform as the harmonics obtained from the randomised connectome do not display clear spatial patterns.

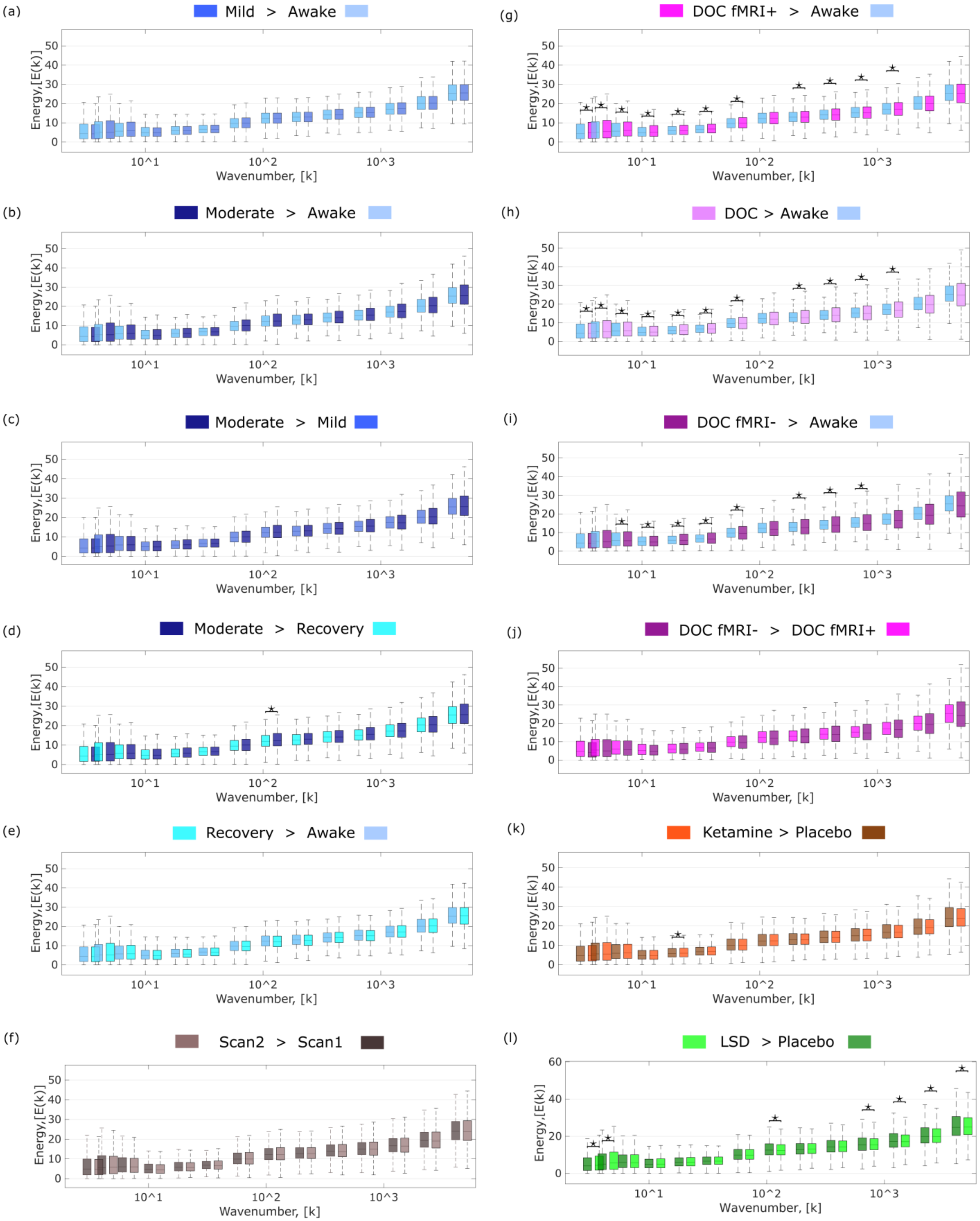

(a)
Mild > Awake
(b)
Moderate > Awake
(c)
Moderate > Mild
(d)
Moderate > Recovery
(e)
Recovery > Awake
(f)
Scan2 > Scan1
(g)
DOC fMRI+ > Awake
(h)
DOC > Awake
(i)
DOC fMRI- > Awake
(j)
DOC fMRI- > DOC fMRI+
(k)
Ketamine > Placebo
(l)
LSD > Placebo
Energy,[E(k)]
Wavenumber, [k]
10^1
10^2
10^3

**Supplementary Figure 16**. **Distribution of frequency-specific energy changes across states of consciousness, for the randomised connectome.** (a) Mild propofol sedation (n=15 subjects with 145 timepoints each) vs wakefulness (n=15 subjects with 145 timepoints each). (b) Moderate anaesthesia (n=15 subjects with 145 timepoints each) vs wakefulness (n=15 subjects with 145 timepoints each). (c) Moderate anaesthesia (n=15 subjects with 145 timepoints each) vs mild sedation (n=15 subjects with 145 timepoints each). (d) Moderate anaesthesia (n=15 subjects with 145 timepoints each) vs post-anaesthetic recovery (n=15 subjects with 145 timepoints each). (e) Recovery (n=15 subjects with 145 timepoints each) vs wakefulness (n=15 subjects with 145 timepoints each). (f) Test-retest scan 2 (n=18 subjects with 155 timepoints each) vs scan 1 (n=18 subjects with 155 timepoints each); (g) DOC patients (n=22 subjects with 295 timepoints each) vs awake healthy controls (n=15 subjects with 145 timepoints each). (h) DOC fMRI+ patients (n=8 subjects with 295 timepoints each) vs awake healthy controls (n=15 subjects with 145 timepoints each). (i) DOC fMRI- patients (n=14 subjects with 295 timepoints each) vs awake healthy controls (n=15 subjects with 145 timepoints each). (j) fMRI- DOC patients (n=14 subjects with 295 timepoints each) vs fMRI+ DOC patients (n=8 subjects with 295 timepoints each). (k) Ketamine (n=20 subjects with 295 timepoints each) vs placebo (n=20 subjects with 295 timepoints each). (l) LSD (n=15 subjects with 435 timepoints each) vs placebo (n=15 subjects with 435 timepoints each). Pairs of conditions (states of consciousness) were compared with linear mixed effects modelling, by treating condition as a fixed effect and subjects as random effects. Timepoints were also included as random effects, nested within subjects. Boxplots display data distributions across subjects and timepoints, for each condition: central line indicates the median; box edges indicate the 25th and 75th percentiles; whiskers: 1.5x inter-quartile interval. * $p < 0.05$, FDR-corrected across 14 frequency bins.

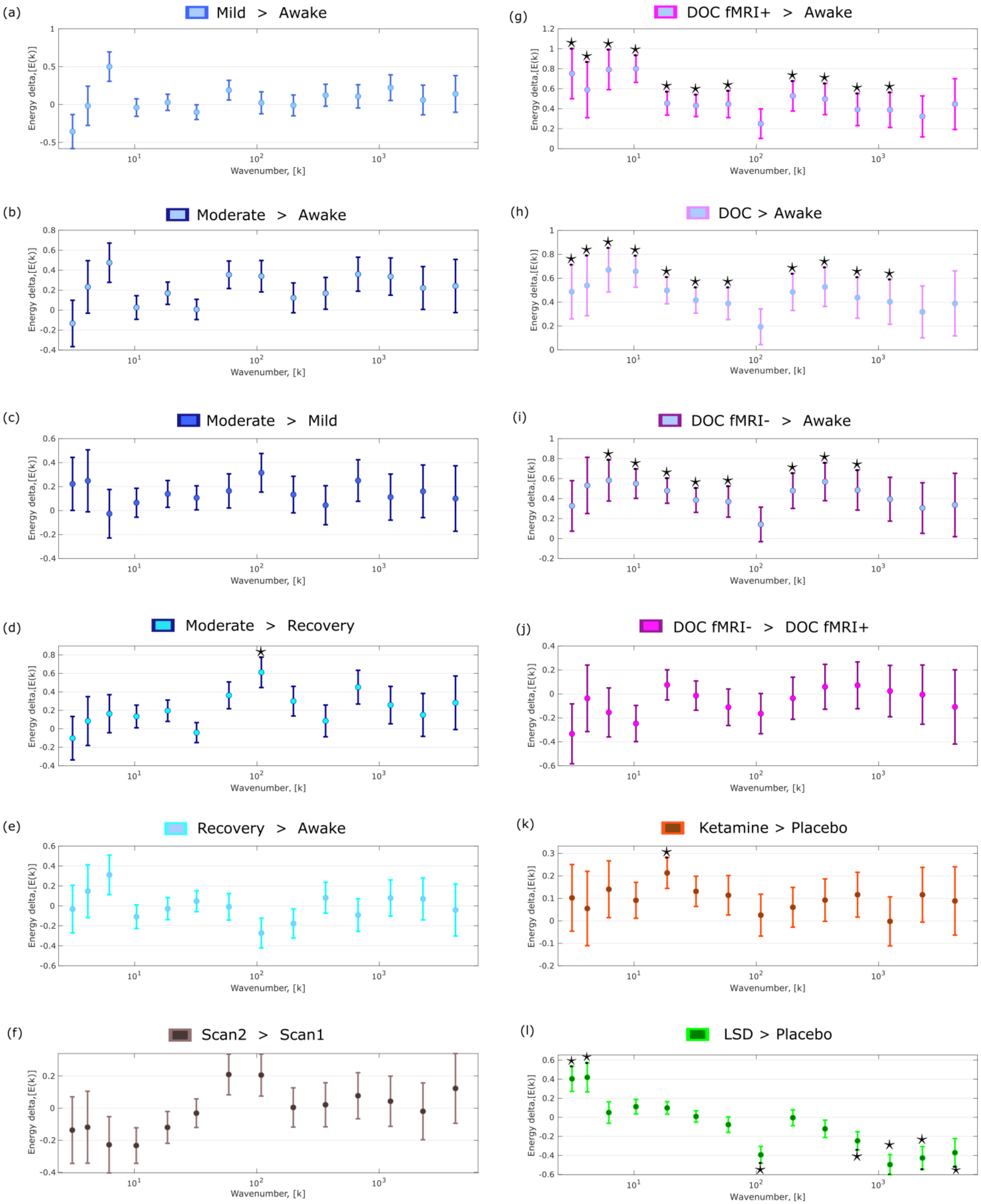
(a)
Mild > Awake
(b)
Moderate > Awake
(c)
Moderate > Mild
(d)
Moderate > Recovery
(e)
Recovery > Awake
(f)
Scan2 > Scan1
(g)
DOC fMRI+ > Awake
(h)
DOC > Awake
(i)
DOC fMRI- > Awake
(j)
DOC fMRI- > DOC fMRI+
(k)
Ketamine > Placebo
(l)
LSD > Placebo
Energy delta,[E(k)]
Wavenumber, [k]
$10^1$
$10^2$
$10^3$

**Supplementary Figure 17. No consistent frequency-specific energy changes are observed across states of consciousness, for the randomised connectome.** Plots show the statistical estimates (fixed effect of condition) and 95% CIs from linear mixed effects modelling between pairs of conditions (states of consciousness), treating condition as a fixed effect and subjects as random effects. Timepoints were also included as random effects, nested within subjects. (a) Mild propofol sedation (n=15 subjects with 145 timepoints each) > wakefulness (n=15 subjects with 145 timepoints each). (b) Moderate anaesthesia (n=15 subjects with 145 timepoints each) > wakefulness (n=15 subjects with 145 timepoints each). (c) Moderate anaesthesia (n=15 subjects with 145 timepoints each) > mild sedation (n=15 subjects with 145 timepoints each). (d) Moderate anaesthesia (n=15 subjects with 145 timepoints each) > post-anaesthetic recovery (n=15 subjects with 145 timepoints each). (e) Recovery (n=15 subjects with 145 timepoints each) > wakefulness (n=15 subjects with 145 timepoints each). (f) Test-retest scan 2 (n=18 subjects with 155 timepoints each) > scan 1 (n=18 subjects with 155 timepoints each); (g) DOC patients (n=22 subjects with 295 timepoints each) > awake healthy controls (n=15 subjects with 145 timepoints each). (h) DOC fMRI+ patients (n=8 subjects with 295 timepoints each) > awake healthy controls (n=15 subjects with 145 timepoints each). (i) DOC fMRI- patients (n=14 subjects with 295 timepoints each) > awake healthy controls (n=15 subjects with 145 timepoints each). (j) fMRI- DOC patients (n=14 subjects with 295 timepoints each) > fMRI+ DOC patients (n=8 subjects with 295 timepoints each). (k) Ketamine (n=20 subjects with 295 timepoints each) > placebo (n=20 subjects with 295 timepoints each). (l) LSD (n=15 subjects with 435 timepoints each) vs placebo (n=15 subjects with 435 timepoints each). * $p < 0.05$, FDR-corrected across 14 logarithmically spaced frequency bins.

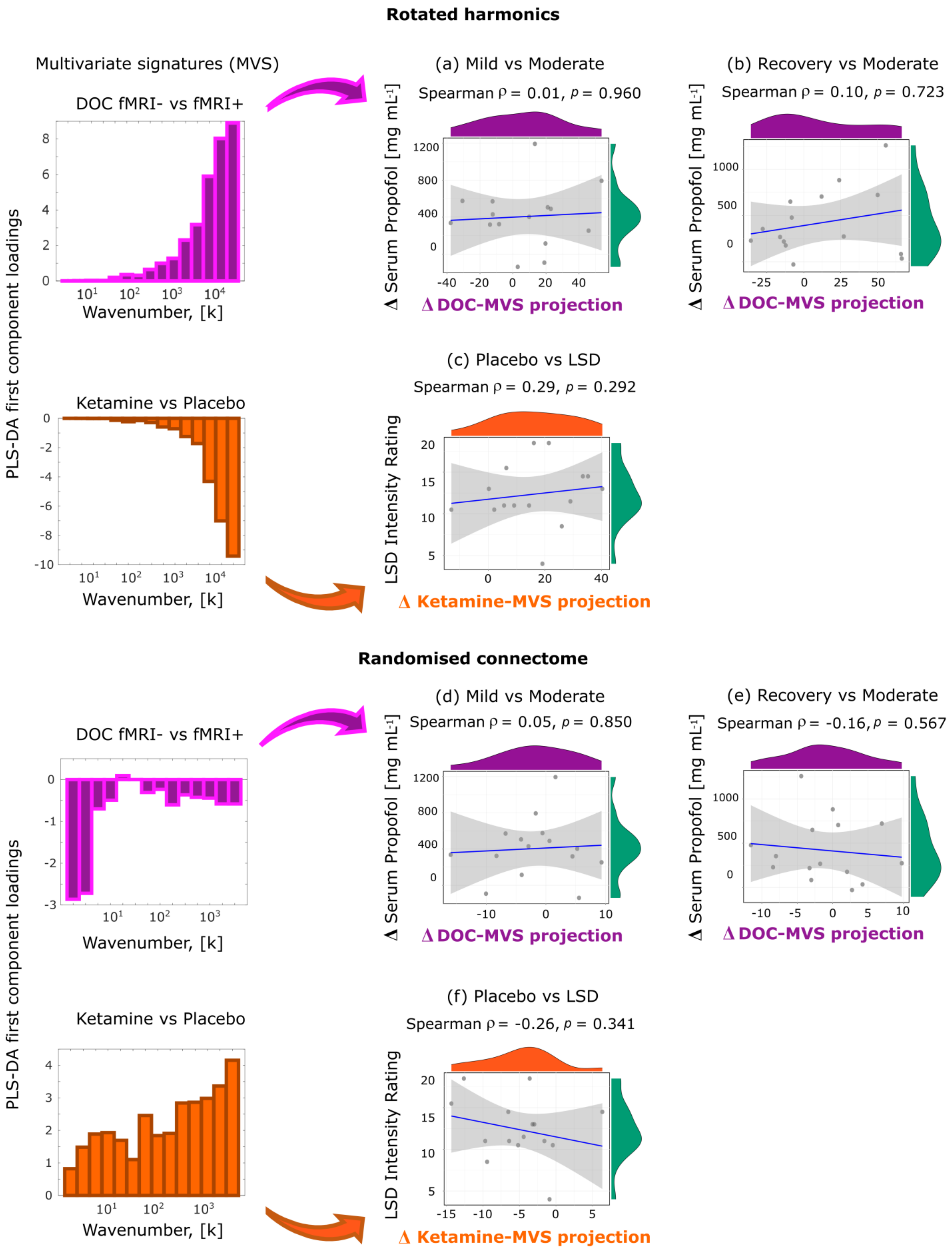
Rotated harmonics
Multivariate signatures (MVS)
DOC fMRI- vs fMRI+
Ketamine vs Placebo
PLS-DA first component loadings
Wavenumber, [k]
(a) Mild vs Moderate
Spearman ρ = 0.01, p = 0.960
(b) Recovery vs Moderate
Spearman ρ = 0.10, p = 0.723
Δ Serum Propofol [mg mL-1]
Δ DOC-MVS projection
(c) Placebo vs LSD
Spearman ρ = 0.29, p = 0.292
LSD Intensity Rating
Δ Ketamine-MVS projection
Randomised connectome
DOC fMRI- vs fMRI+
Ketamine vs Placebo
PLS-DA first component loadings
Wavenumber, [k]
(d) Mild vs Moderate
Spearman ρ = 0.05, p = 0.850
(e) Recovery vs Moderate
Spearman ρ = -0.16, p = 0.567
Δ Serum Propofol [mg mL-1]
Δ DOC-MVS projection
(f) Placebo vs LSD
Spearman ρ = -0.26, p = 0.341
LSD Intensity Rating
Δ Ketamine-MVS projection

**Supplementary Figure 18. No generalisation of connectome harmonic signatures is observed when using rotated harmonics or a randomised connectome.** For (a-c), the connectome harmonic energy spectrum is obtained from rotated harmonics. (a) Scatterplot of the change (moderate anaesthesia minus mild; n=15 subjects) in connectome harmonic energy projection onto the multivariate energy signature (MVS) derived from the DOC dataset, versus the change in propofol levels in volunteers' blood serum, between mild and moderate propofol anaesthesia. (b) Scatterplot of the change (moderate minus recovery; n=15 subjects) in connectome harmonic energy projection onto the multivariate signature derived from the DOC dataset, versus the change in propofol levels in volunteers' blood serum, between moderate anaesthesia and recovery. (c) Scatterplot of the change (LSD minus placebo; n=15 subjects) in connectome harmonic energy projection onto the multivariate signature derived from the ketamine dataset, versus the subjective intensity of the psychedelic experience induced by LSD. For (d-f), the connectome harmonic energy spectrum is obtained from the harmonics of a randomised connectome. (d) Scatterplot of the change (moderate anaesthesia minus mild; n=15 subjects) in connectome harmonic energy projection onto the multivariate energy signature (MVS) derived from the DOC dataset, versus the change in propofol levels in volunteers' blood serum, between mild and moderate propofol anaesthesia. (e) Scatterplot of the change (moderate minus recovery; n=15 subjects) in connectome harmonic energy projection onto the multivariate signature derived from the DOC dataset, versus the change in propofol levels in volunteers' blood serum, between moderate anaesthesia and recovery. (f) Scatterplot of the change (LSD minus placebo; n=15 subjects) in connectome harmonic energy projection onto the multivariate signature derived from the ketamine dataset, versus the subjective intensity of the psychedelic experience induced by LSD. Shading indicates 95% confidence interval.

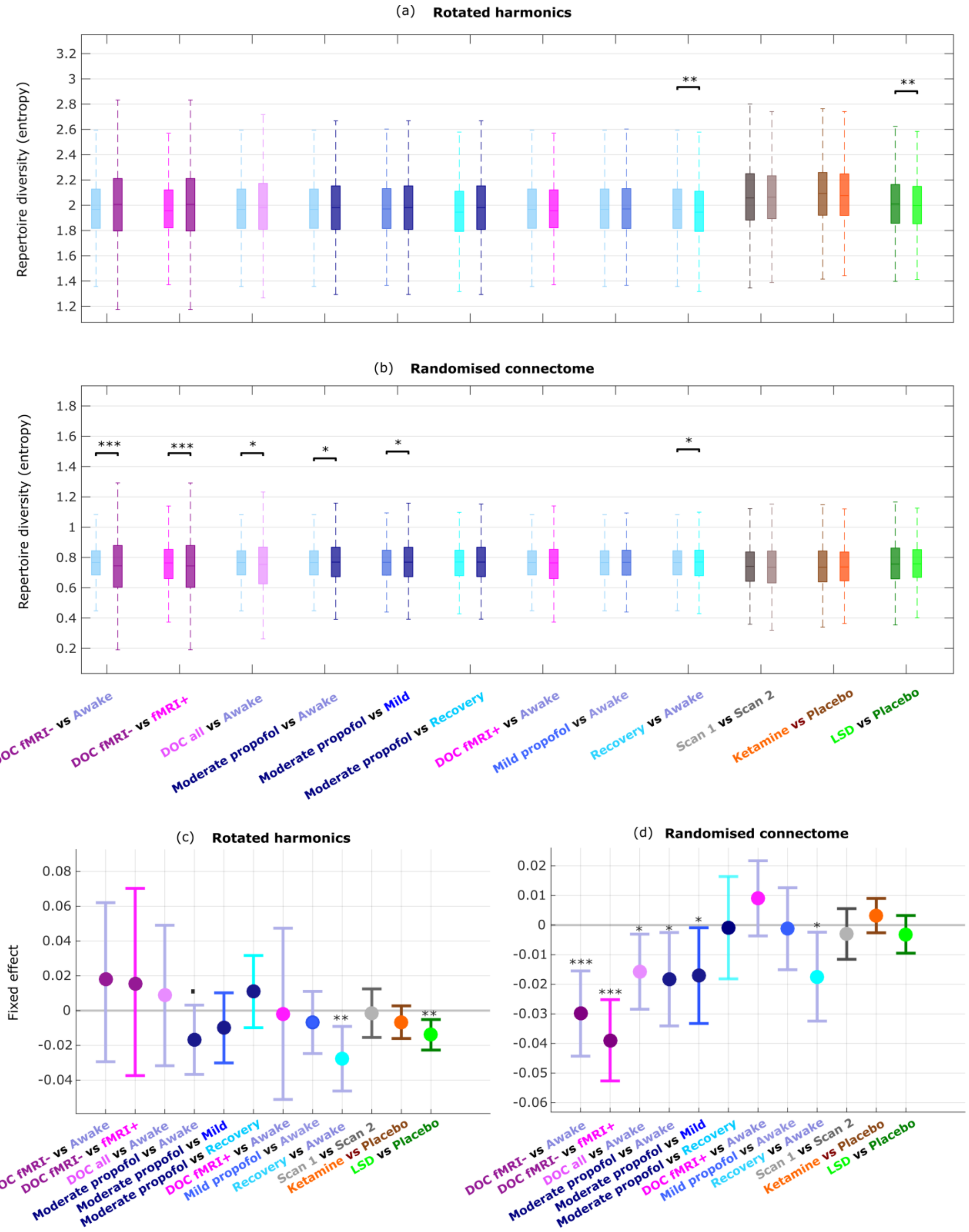


**Supplementary Figure 19. Repertoire diversity does not track level of consciousness across datasets, when obtained from rotated harmonics or from a randomised connectome.** (a) Distribution of diversity of connectome harmonic repertoire obtained from rotated harmonics. (b) Distribution of diversity of connectome harmonic repertoire obtained from a randomised connectome. Boxplots display data distributions across subjects and timepoints, for each condition: central line indicates the median; box edges indicate the 25th and 75th percentiles; whiskers: 1.5x inter-quartile interval. (c-d) Plots show the statistical estimates (fixed effect of condition) and 95% CIs for the difference in diversity of connectome harmonic repertoire between pairs of conditions (states of consciousness), from linear mixed effects modelling treating condition as a fixed effect and subjects as random effects. Timepoints were also included as random effects, nested within subjects. *** $p < 0.001$; ** $p < 0.01$; * $p < 0.05$; · $p < 0.10$. Wakefulness, mild sedation, moderate anaesthesia, and recovery: n=15 subjects with 145 timepoints each, for each condition. DOC fMRI+ patients: n=8 subjects with 295 timepoints each; DOC fMRI- patients: n=14 subjects with 295 timepoints each. Test-retest scan 1 and scan 2: n=18 subjects with 155 timepoints each, for each condition. Ketamine and placebo: n=20 subjects with 295 timepoints each, for each condition. LSD and placebo: n=15 subjects with 435 timepoints each, for each condition.

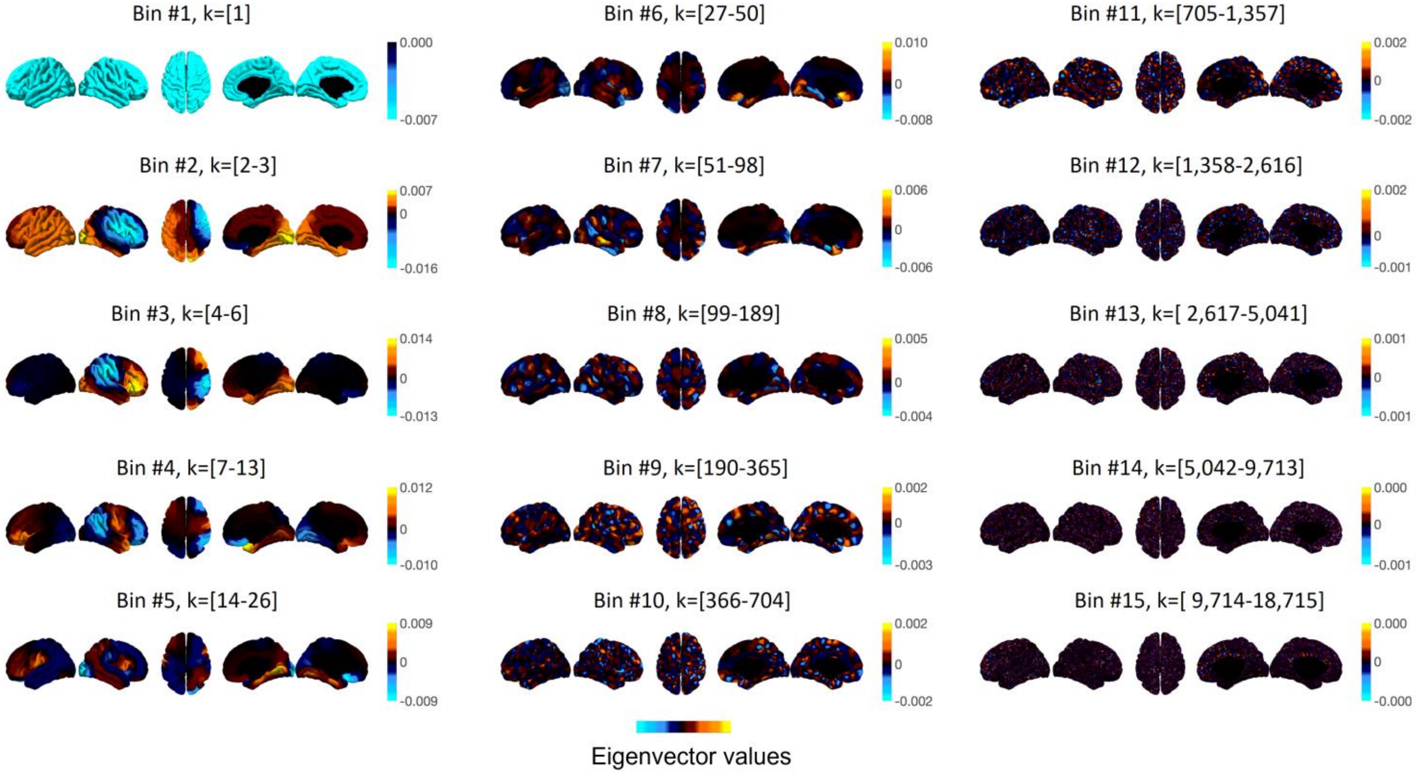


**Supplementary Figure 20**. **Binned connectome harmonics for the HCP-985 connectome**. Surface projections of connectome harmonics averaged over each of 15 logarithmically spaced bins (with corresponding wavenumbers *k* indicated in braces), showing the progressive increase in complexity and granularity of the connectome harmonic patterns, with increasing spatial frequency. Note that only the first 14 bins are used for analysis.

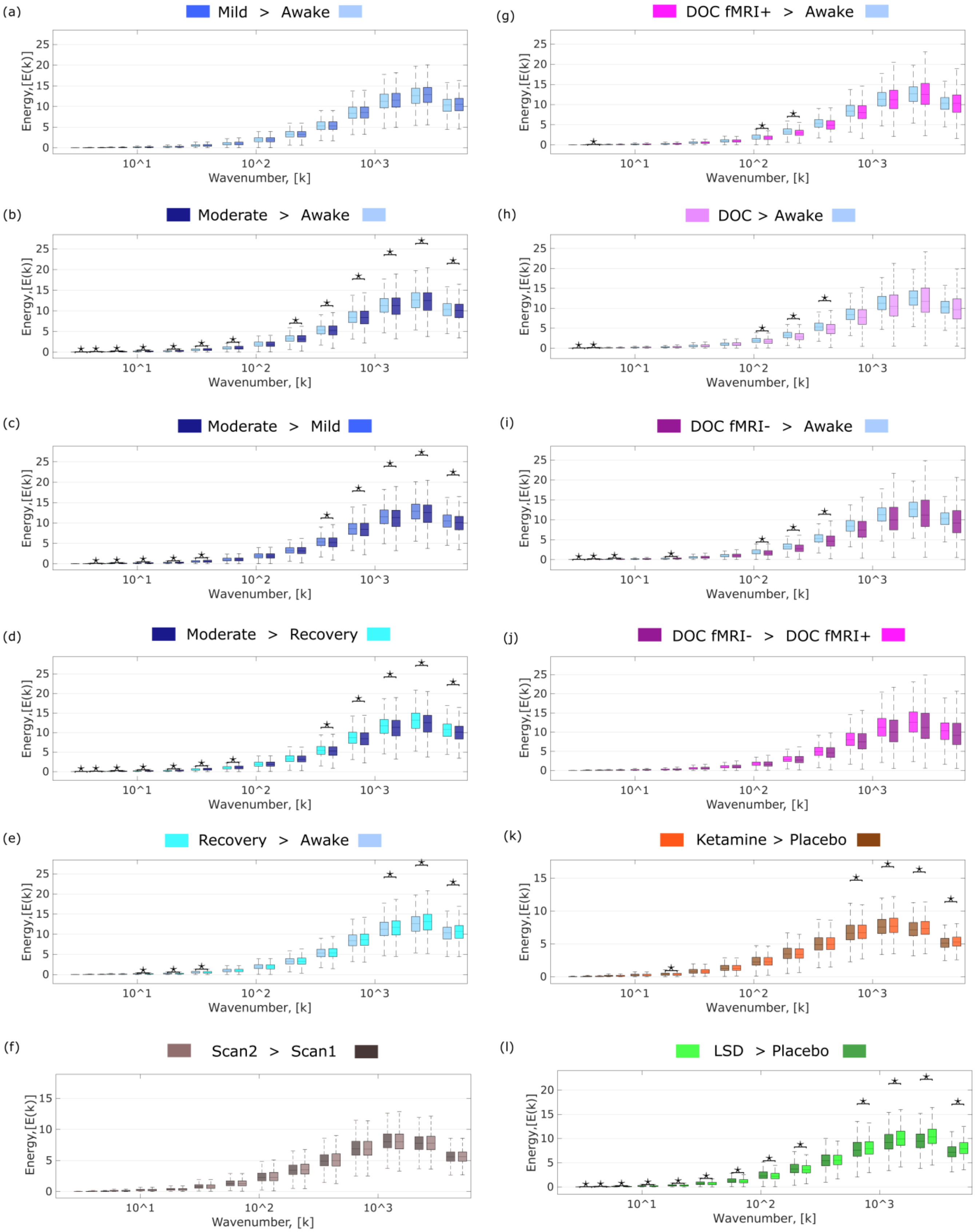
(a)
Mild > Awake
(b)
Moderate > Awake
(c)
Moderate > Mild
(d)
Moderate > Recovery
(e)
Recovery > Awake
(f)
Scan2 > Scan1
(g)
DOC fMRI+ > Awake
(h)
DOC > Awake
(i)
DOC fMRI- > Awake
(j)
DOC fMRI- > DOC fMRI+
(k)
Ketamine > Placebo
(l)
LSD > Placebo
Energy,[E(k)]
Wavenumber, [k]
10^1
10^2
10^3

**Supplementary Figure 21**. **Distribution of frequency-specific energy across states of consciousness, for the HCP-985 connectome.** (a) Mild propofol sedation (n=15 subjects with 145 timepoints each) vs wakefulness (n=15 subjects with 145 timepoints each). (b) Moderate anaesthesia (n=15 subjects with 145 timepoints each) vs wakefulness (n=15 subjects with 145 timepoints each). (c) Moderate anaesthesia (n=15 subjects with 145 timepoints each) vs mild sedation (n=15 subjects with 145 timepoints each). (d) Moderate anaesthesia (n=15 subjects with 145 timepoints each) vs post-anaesthetic recovery (n=15 subjects with 145 timepoints each). (e) Recovery (n=15 subjects with 145 timepoints each) vs wakefulness (n=15 subjects with 145 timepoints each). (f) Test-retest scan 2 (n=18 subjects with 155 timepoints each) vs scan 1 (n=18 subjects with 155 timepoints each); (g) DOC patients (n=22 subjects with 295 timepoints each) vs awake healthy controls (n=15 subjects with 145 timepoints each). (h) DOC fMRI+ patients (n=8 subjects with 295 timepoints each) vs awake healthy controls (n=15 subjects with 145 timepoints each). (i) DOC fMRI- patients (n=14 subjects with 295 timepoints each) vs awake healthy controls (n=15 subjects with 145 timepoints each). (j) fMRI- DOC patients (n=14 subjects with 295 timepoints each) vs fMRI+ DOC patients (n=8 subjects with 295 timepoints each). (k) Ketamine (n=20 subjects with 295 timepoints each) vs placebo (n=20 subjects with 295 timepoints each). (l) LSD (n=15 subjects with 435 timepoints each) vs placebo (n=15 subjects with 435 timepoints each). Pairs of conditions (states of consciousness) were compared with linear mixed effects modelling, by treating condition as a fixed effect and subjects as random effects. Timepoints were also included as random effects, nested within subjects. Boxplots display data distributions across subjects and timepoints, for each condition: central line indicates the median; box edges indicate the 25th and 75th percentiles; whiskers: 1.5x inter-quartile interval. * $p < 0.05$, FDR-corrected across 14 frequency bins.

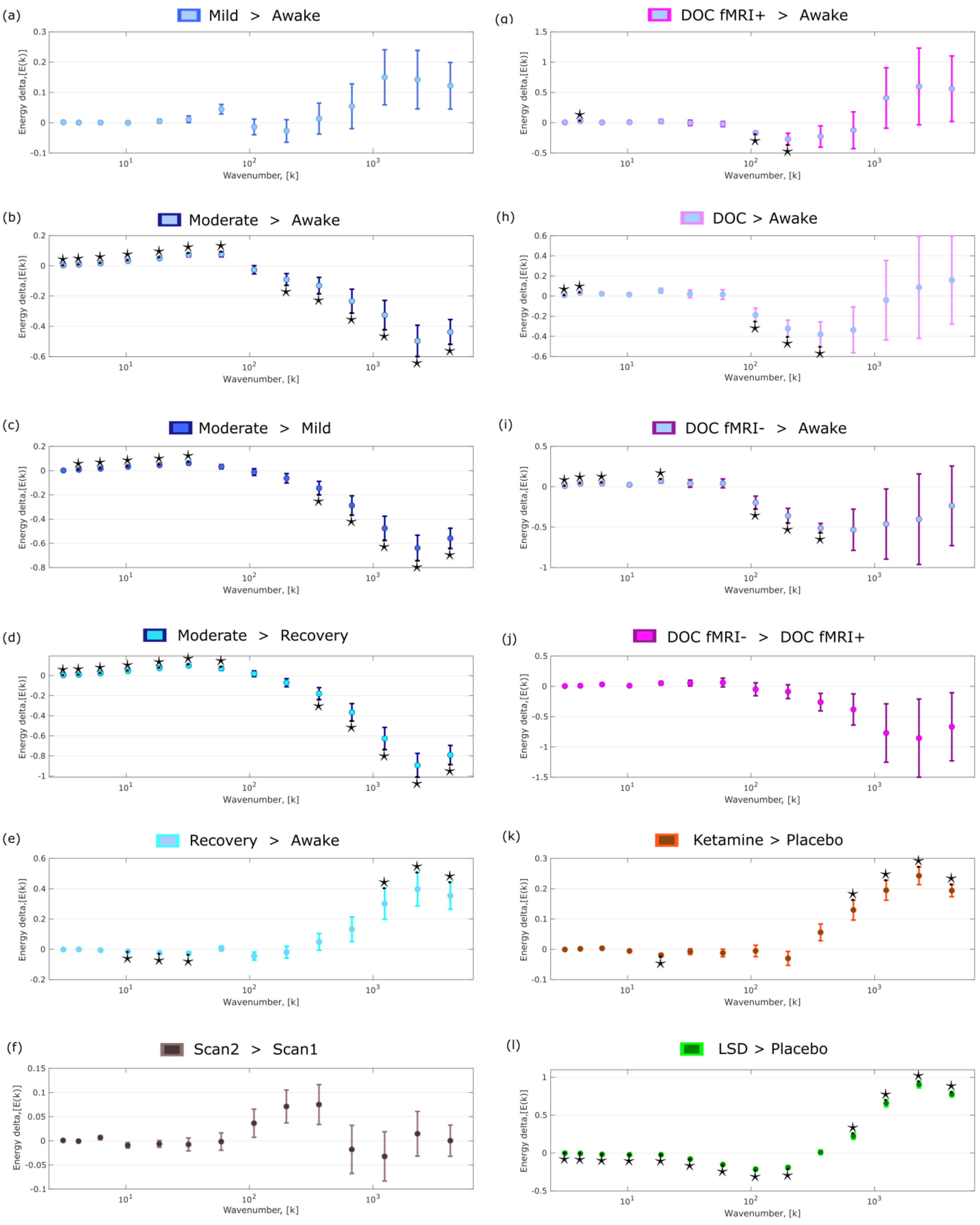
(a)
Mild > Awake
(b)
Moderate > Awake
(c)
Moderate > Mild
(d)
Moderate > Recovery
(e)
Recovery > Awake
(f)
Scan2 > Scan1
(g)
DOC fMRI+ > Awake
(h)
DOC > Awake
(i)
DOC fMRI- > Awake
(j)
DOC fMRI- > DOC fMRI+
(k)
Ketamine > Placebo
(l)
LSD > Placebo
Energy delta,[E(k)]
Wavenumber, [k]

**Supplementary Figure 22**. **Differences of frequency-specific energy across states of consciousness, for the HCP-985 connectome.** Plots show the statistical estimates (fixed effect of condition) and 95% CIs from linear mixed effects modelling between pairs of conditions (states of consciousness), treating condition as a fixed effect and subjects as random effects. Timepoints were also included as random effects, nested within subjects. (a) Mild propofol sedation (n=15 subjects with 145 timepoints each) > wakefulness (n=15 subjects with 145 timepoints each). (b) Moderate anaesthesia (n=15 subjects with 145 timepoints each) > wakefulness (n=15 subjects with 145 timepoints each). (c) Moderate anaesthesia (n=15 subjects with 145 timepoints each) > mild sedation (n=15 subjects with 145 timepoints each). (d) Moderate anaesthesia (n=15 subjects with 145 timepoints each) > post-anaesthetic recovery (n=15 subjects with 145 timepoints each). (e) Recovery (n=15 subjects with 145 timepoints each) > wakefulness (n=15 subjects with 145 timepoints each). (f) Test-retest scan 2 (n=18 subjects with 155 timepoints each) > scan 1 (n=18 subjects with 155 timepoints each); (g) DOC patients (n=22 subjects with 295 timepoints each) > awake healthy controls (n=15 subjects with 145 timepoints each). (h) DOC fMRI+ patients (n=8 subjects with 295 timepoints each) > awake healthy controls (n=15 subjects with 145 timepoints each). (i) DOC fMRI- patients (n=14 subjects with 295 timepoints each) > awake healthy controls (n=15 subjects with 145 timepoints each). (j) fMRI- DOC patients (n=14 subjects with 295 timepoints each) > fMRI+ DOC patients (n=8 subjects with 295 timepoints each). (k) Ketamine (n=20 subjects with 295 timepoints each) > placebo (n=20 subjects with 295 timepoints each). (l) LSD (n=15 subjects with 435 timepoints each) vs placebo (n=15 subjects with 435 timepoints each). * $p < 0.05$, FDR-corrected across 14 logarithmically spaced frequency bins.

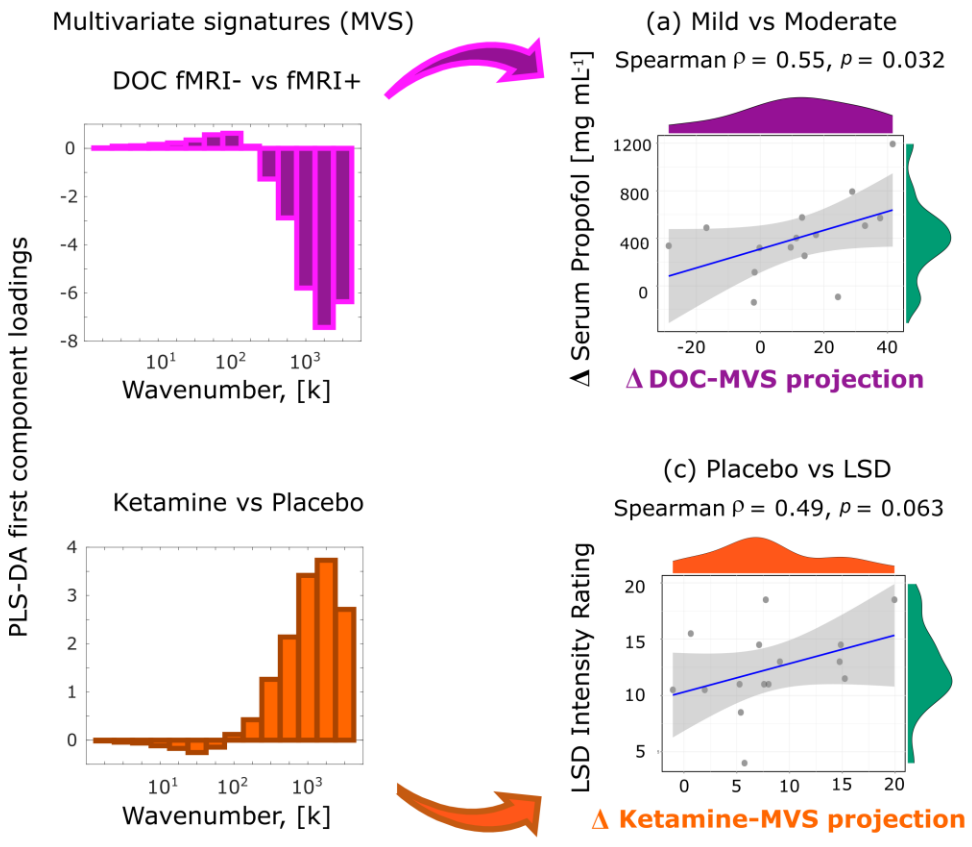


**Supplementary Figure 23. Correlations between multivariate energy signatures (MVS) and propofol levels and LSD intensity scores, are replicated with the HCP-985 connectome.** (a) Scatterplot of the change (moderate anaesthesia minus mild; n=15 subjects) in connectome harmonic energy projection onto the multivariate energy signature (MVS) derived from the DOC dataset, versus the change in propofol levels in volunteers' blood serum, between mild and moderate propofol anaesthesia. (b) Scatterplot of the change (moderate minus recovery; n=15 subjects) in connectome harmonic energy projection onto the multivariate signature derived from the DOC dataset, versus the change in propofol levels in volunteers' blood serum, between moderate anaesthesia and recovery. (c) Scatterplot of the change (LSD minus placebo; n=15 subjects) in connectome harmonic energy projection onto the multivariate signature derived from the ketamine dataset, versus the subjective intensity of the psychedelic experience induced by LSD. Shading indicates 95% confidence interval.

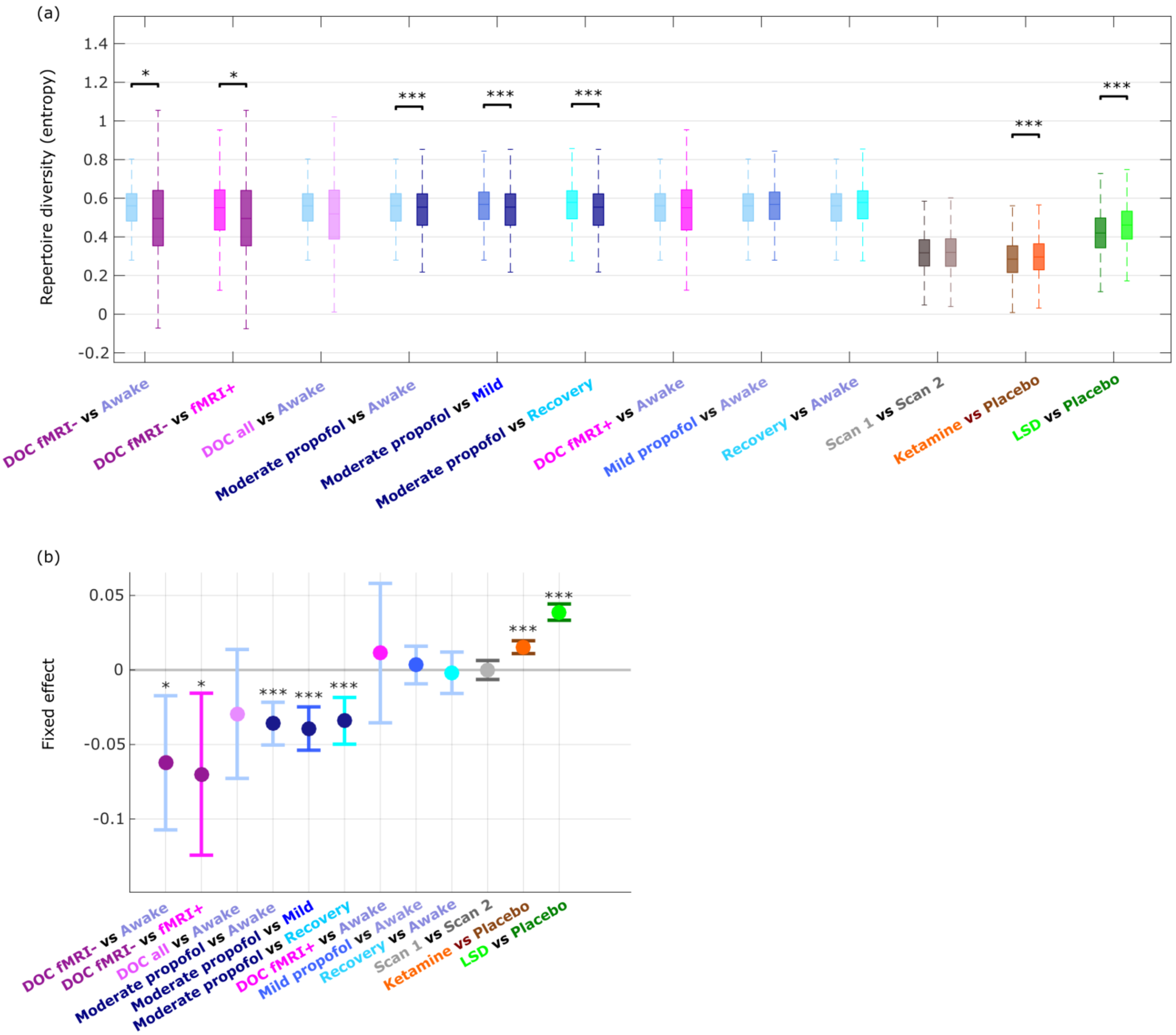


**Supplementary Figure 24. Replication of repertoire diversity results for the HCP-985 connectome.** (a) Distribution of diversity of connectome harmonic repertoire. Boxplots display data distributions across subjects and timepoints, for each condition: central line indicates the median; box edges indicate the 25th and 75th percentiles; whiskers: 1.5x inter-quartile interval. (b) Plots show the statistical estimates (fixed effect of condition) and 95% CIs for the difference in diversity of connectome harmonic repertoire between pairs of conditions (states of consciousness), from linear mixed effects modelling treating condition as a fixed effect and subjects as random effects. Timepoints were also included as random effects, nested within subjects. *** $p < 0.001$; * $p < 0.05$; $^{\cdot}$ $p < 0.10$. Wakefulness, mild sedation, moderate anaesthesia, and recovery: n=15 subjects with 145 timepoints each, for each condition. DOC fMRI+ patients: n=8 subjects with 295 timepoints each; DOC fMRI- patients: n=14 subjects with 295 timepoints each. Test-retest scan 1 and scan 2: n=18 subjects with 155 timepoints each, for each condition. Ketamine and placebo: n=20 subjects with 295 timepoints each, for each condition. LSD and placebo: n=15 subjects with 435 timepoints each, for each condition.

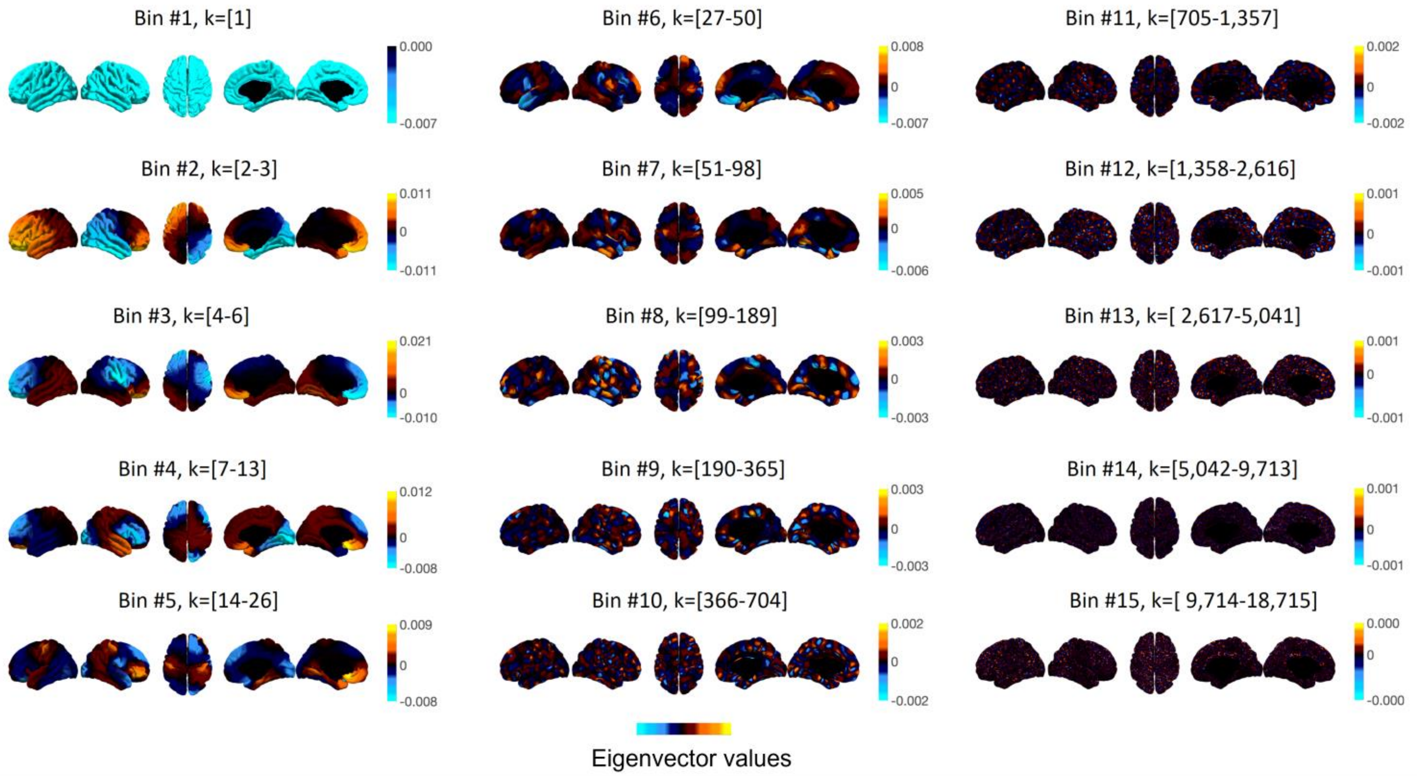


**Supplementary Figure 25**. **Binned connectome harmonics for the MGH-32 connectome**. Surface projections of connectome harmonics averaged over each of 15 logarithmically spaced bins (with corresponding wavenumbers *k* indicated in braces), showing the progressive increase in complexity and granularity of the connectome harmonic patterns, with increasing spatial frequency. Note that only the first 14 bins are used for analysis.

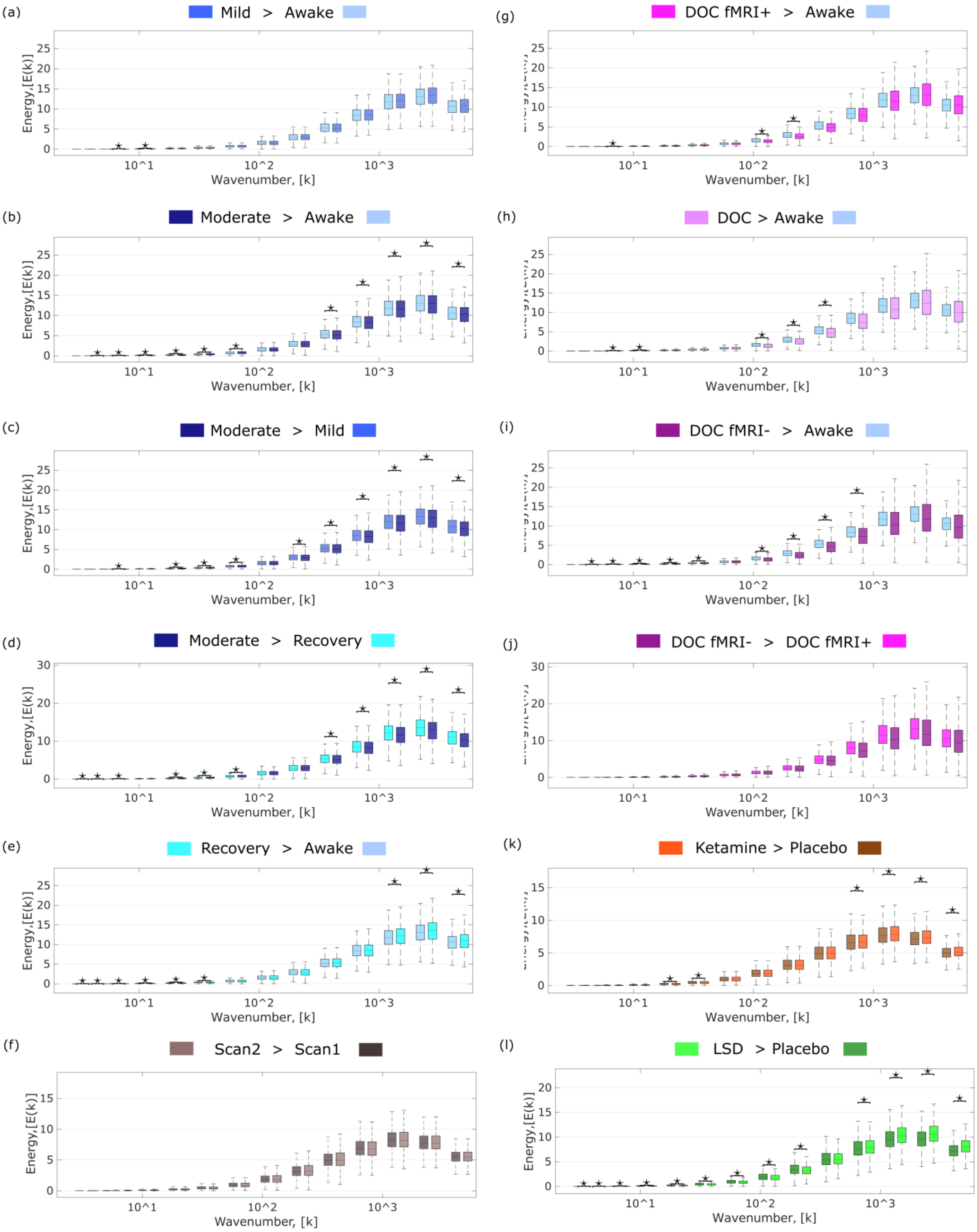
(a)
Mild > Awake
Energy,[E(k)]
Wavenumber, [k]
(b)
Moderate > Awake
(c)
Moderate > Mild
(d)
Moderate > Recovery
(e)
Recovery > Awake
(f)
Scan2 > Scan1
(g)
DOC fMRI+ > Awake
(h)
DOC > Awake
(i)
DOC fMRI- > Awake
(j)
DOC fMRI- > DOC fMRI+
(k)
Ketamine > Placebo
(l)
LSD > Placebo
10^1
10^2
10^3

**Supplementary Figure 26**. **Distribution of frequency-specific energy across states of consciousness, for the MGH-32 connectome.** (a) Mild propofol sedation (n=15 subjects with 145 timepoints each) vs wakefulness (n=15 subjects with 145 timepoints each). (b) Moderate anaesthesia (n=15 subjects with 145 timepoints each) vs wakefulness (n=15 subjects with 145 timepoints each). (c) Moderate anaesthesia (n=15 subjects with 145 timepoints each) vs mild sedation (n=15 subjects with 145 timepoints each). (d) Moderate anaesthesia (n=15 subjects with 145 timepoints each) vs post-anaesthetic recovery (n=15 subjects with 145 timepoints each). (e) Recovery (n=15 subjects with 145 timepoints each) vs wakefulness (n=15 subjects with 145 timepoints each). (f) Test-retest scan 2 (n=18 subjects with 155 timepoints each) vs scan 1 (n=18 subjects with 155 timepoints each); (g) DOC patients (n=22 subjects with 295 timepoints each) vs awake healthy controls (n=15 subjects with 145 timepoints each). (h) DOC fMRI+ patients (n=8 subjects with 295 timepoints each) vs awake healthy controls (n=15 subjects with 145 timepoints each). (i) DOC fMRI- patients (n=14 subjects with 295 timepoints each) vs awake healthy controls (n=15 subjects with 145 timepoints each). (j) fMRI- DOC patients (n=14 subjects with 295 timepoints each) vs fMRI+ DOC patients (n=8 subjects with 295 timepoints each). (k) Ketamine (n=20 subjects with 295 timepoints each) vs placebo (n=20 subjects with 295 timepoints each). (l) LSD (n=15 subjects with 435 timepoints each) vs placebo (n=15 subjects with 435 timepoints each). Pairs of conditions (states of consciousness) were compared with linear mixed effects modelling, by treating condition as a fixed effect and subjects as random effects. Timepoints were also included as random effects, nested within subjects. Boxplots display data distributions across subjects and timepoints, for each condition: central line indicates the median; box edges indicate the 25th and 75th percentiles; whiskers: 1.5x inter-quartile interval. * $p < 0.05$, FDR-corrected across 14 frequency bins.

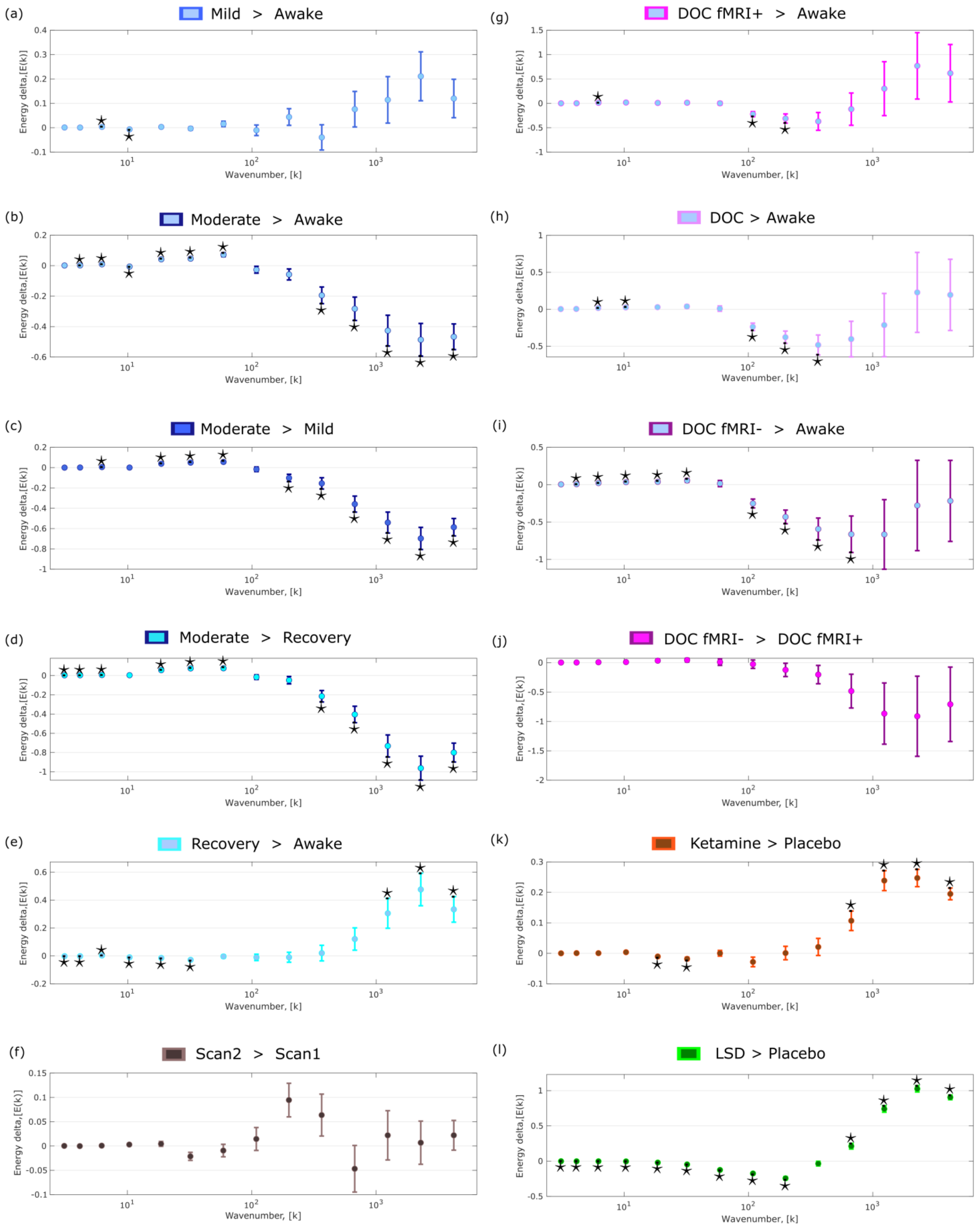
(a)
Mild > Awake
(b)
Moderate > Awake
(c)
Moderate > Mild
(d)
Moderate > Recovery
(e)
Recovery > Awake
(f)
Scan2 > Scan1
(g)
DOC fMRI+ > Awake
(h)
DOC > Awake
(i)
DOC fMRI- > Awake
(j)
DOC fMRI- > DOC fMRI+
(k)
Ketamine > Placebo
(l)
LSD > Placebo
Energy delta,[E(k)]
Wavenumber, [k]

**Supplementary Figure 27**. **Differences of frequency-specific energy across states of consciousness, for the MGH-32 connectome.** Plots show the statistical estimates (fixed effect of condition) and 95% CIs from linear mixed effects modelling between pairs of conditions (states of consciousness), treating condition as a fixed effect and subjects as random effects. Timepoints were also included as random effects, nested within subjects. (a) Mild propofol sedation (n=15 subjects with 145 timepoints each) > wakefulness (n=15 subjects with 145 timepoints each). (b) Moderate anaesthesia (n=15 subjects with 145 timepoints each) > wakefulness (n=15 subjects with 145 timepoints each). (c) Moderate anaesthesia (n=15 subjects with 145 timepoints each) > mild sedation (n=15 subjects with 145 timepoints each). (d) Moderate anaesthesia (n=15 subjects with 145 timepoints each) > post-anaesthetic recovery (n=15 subjects with 145 timepoints each). (e) Recovery (n=15 subjects with 145 timepoints each) > wakefulness (n=15 subjects with 145 timepoints each). (f) Test-retest scan 2 (n=18 subjects with 155 timepoints each) > scan 1 (n=18 subjects with 155 timepoints each); (g) DOC patients (n=22 subjects with 295 timepoints each) > awake healthy controls (n=15 subjects with 145 timepoints each). (h) DOC fMRI+ patients (n=8 subjects with 295 timepoints each) > awake healthy controls (n=15 subjects with 145 timepoints each). (i) DOC fMRI- patients (n=14 subjects with 295 timepoints each) > awake healthy controls (n=15 subjects with 145 timepoints each). (j) fMRI- DOC patients (n=14 subjects with 295 timepoints each) > fMRI+ DOC patients (n=8 subjects with 295 timepoints each). (k) Ketamine (n=20 subjects with 295 timepoints each) > placebo (n=20 subjects with 295 timepoints each). (l) LSD (n=15 subjects with 435 timepoints each) vs placebo (n=15 subjects with 435 timepoints each). * $p < 0.05$, FDR-corrected across 14 logarithmically spaced frequency bins.

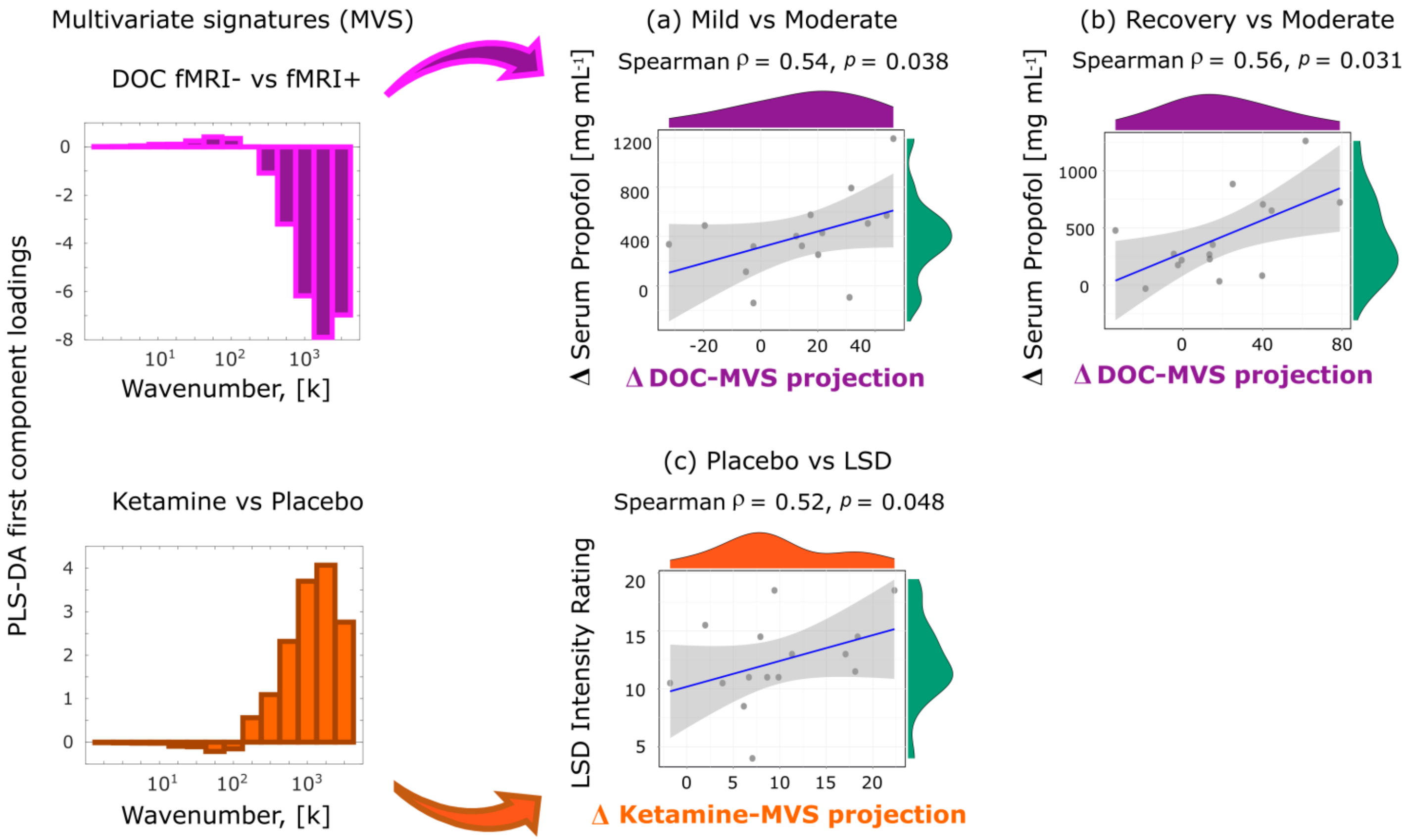


**Supplementary Figure 28. Correlations between multivariate energy signatures (MVS) and propofol levels and LSD intensity scores, are replicated with the MGH-32 connectome.** (a) Scatterplot of the change (moderate anaesthesia minus mild; n=15 subjects) in connectome harmonic energy projection onto the multivariate energy signature (MVS) derived from the DOC dataset, versus the change in propofol levels in volunteers' blood serum, between mild and moderate propofol anaesthesia. (b) Scatterplot of the change (moderate minus recovery; n=15 subjects) in connectome harmonic energy projection onto the multivariate signature derived from the DOC dataset, versus the change in propofol levels in volunteers' blood serum, between moderate anaesthesia and recovery. (c) Scatterplot of the change (LSD minus placebo; n=15 subjects) in connectome harmonic energy projection onto the multivariate signature derived from the ketamine dataset, versus the subjective intensity of the psychedelic experience induced by LSD. Shading indicates 95% confidence interval.

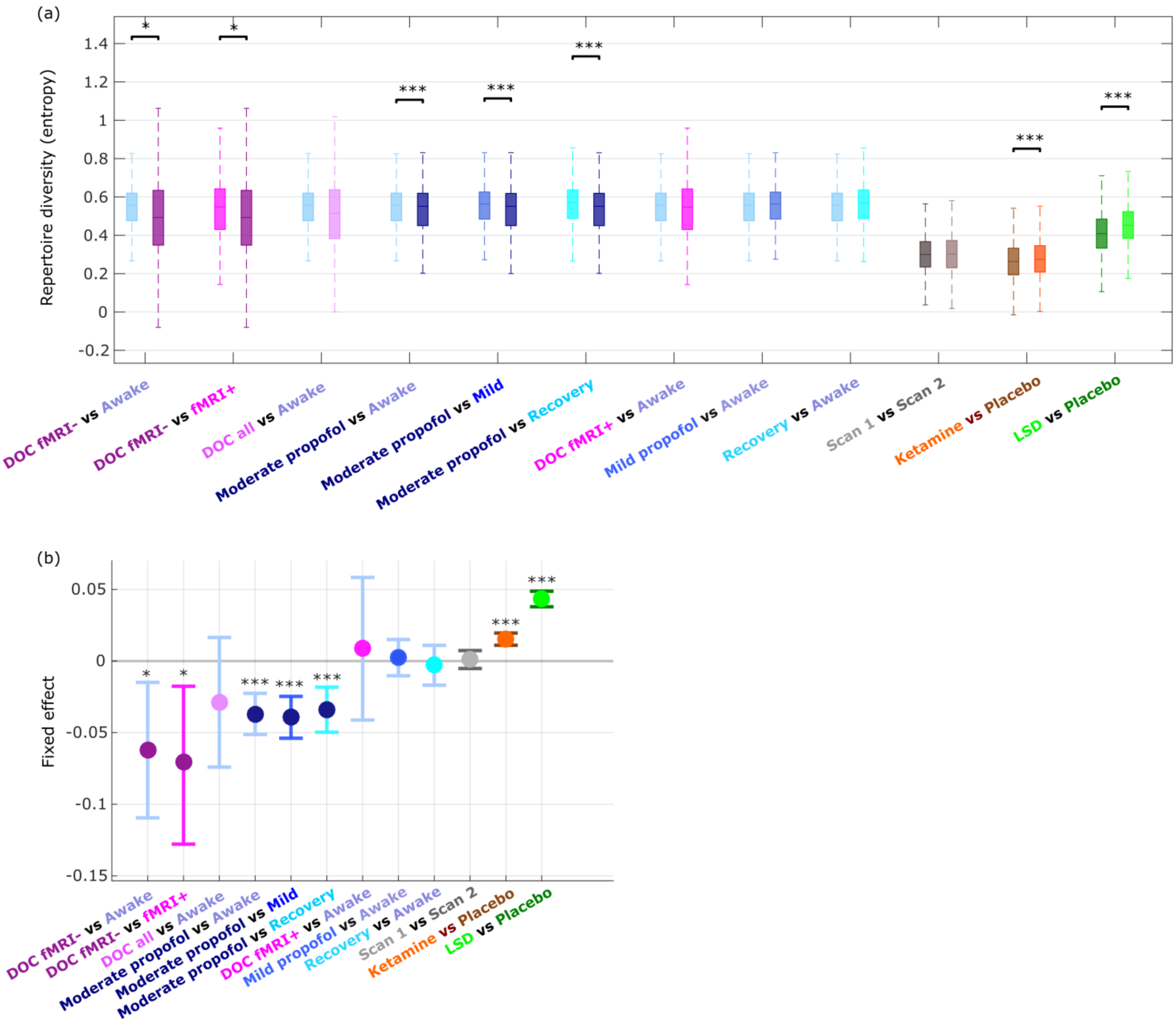


**Supplementary Figure 29. Replication of repertoire diversity results for the MGH-32 connectome.** (a) Distribution of diversity of connectome harmonic repertoire. Boxplots display data distributions across subjects and timepoints, for each condition: central line indicates the median; box edges indicate the 25th and 75th percentiles; whiskers: 1.5x inter-quartile interval. (b) Plots show the statistical estimates (fixed effect of condition) and 95% CIs for the difference in diversity of connectome harmonic repertoire between pairs of conditions (states of consciousness), from linear mixed effects modelling treating condition as a fixed effect and subjects as random effects. Timepoints were also included as random effects, nested within subjects. *** $p < 0.001$; * $p < 0.05$; · $p < 0.10$. Wakefulness, mild sedation, moderate anaesthesia, and recovery: n=15 subjects with 145 timepoints each, for each condition. DOC fMRI+ patients: n=8 subjects with 295 timepoints each; DOC fMRI- patients: n=14 subjects with 295 timepoints each. Test-retest scan 1 and scan 2: n=18 subjects with 155 timepoints each, for each condition. Ketamine and placebo: n=20 subjects with 295 timepoints each, for each condition. LSD and placebo: n=15 subjects with 435 timepoints each, for each condition.

# Supplementary Tables

| Contrast | Fixed Effect | 95% CI Lower | 95% CI Upper | p-value | Significance |
|---|---|---|---|---|---|
| Awake vs fMRI- | -0.068 | -0.127 | -0.009 | 0.024 | * |
| fMRI+ vs fMRI- | -0.056 | -0.127 | 0.014 | 0.117 | n.s. |
| Awake vs DOC | -0.041 | -0.092 | 0.010 | 0.113 | n.s. |
| Awake vs Moderate Propofol | -0.039 | -0.052 | -0.026 | 8.92E-09 | *** |
| Mild vs Moderate Propofol | -0.040 | -0.054 | -0.027 | 5.33E-09 | *** |
| Recovery vs Moderate Propofol | -0.040 | -0.054 | -0.025 | 1.26E-07 | *** |
| Awake vs fMRI+ | -0.009 | -0.059 | 0.042 | 0.738 | n.s. |
| Awake vs Mild Propofol | 0.001 | -0.011 | 0.013 | 0.875 | n.s. |
| Scan 1 vs Scan 2 | 0.001 | -0.013 | 0.014 | 0.926 | n.s. |
| Awake vs Recovery | -0.003 | -0.008 | 0.003 | 0.372 | n.s. |
| Placebo vs Ketamine | 0.020 | 0.016 | 0.024 | p<E-10 | *** |
| Placebo vs LSD | 0.054 | 0.049 | 0.059 | p<E-10 | *** |
| DOC Any response vs No Response | -0.074 | -0.139 | -0.008 | 0.026 | * |

**Supplementary Table 1.** LME results for repertoire diversity of the connectome harmonics. * $p < 0.05$; ** $p < 0.01$; *** $p < 0.001$; ** $p < 0.01$; . $p < 0.10$; n.s. not significant.

| Contrast | Spearman rho | p-value | Significance |
|---|---|---|---|
| **Awake vs fMRI-** | -0.08 | 0.768 | n.s. |
| **fMRI+ vs fMRI-** | 0.12 | 0.662 | n.s. |
| **Awake vs DOC** | -0.24 | 0.384 | n.s. |
| **Awake vs Moderate Propofol** | 0.03 | 0.899 | n.s. |
| **Mild vs Moderate Propofol** | 0.11 | 0.691 | n.s. |
| **Recovery vs Moderate Propofol** | 0.07 | 0.786 | n.s. |
| **Awake vs fMRI+** | -0.33 | 0.230 | n.s. |
| **Awake vs Mild Propofol** | -0.31 | 0.258 | n.s. |
| **Awake vs Recovery** | -0.12 | 0.660 | n.s. |
| **Placebo vs Ketamine** | -0.19 | 0.485 | n.s. |
| **Placebo vs LSD** | -0.15 | 0.590 | n.s. |

**Supplementary Table 2.** Spearman correlation between the connectome harmonic energy signatures obtained from different states of consciousness (Figures 3 and S4), and the signature obtained from comparing test and retest scans from the same awake volunteers (Supplementary Figure 2). n.s. not significant.

| Contrast | Fixed Effect | 95% CI Lower | 95% CI Upper | p-value | Significance |
|---|---|---|---|---|---|
| **Awake vs fMRI-** | 0.016 | -0.031 | 0.062 | 0.510 | n.s. |
| **fMRI+ vs fMRI-** | 0.015 | -0.038 | 0.068 | 0.580 | n.s. |
| **Awake vs DOC** | 0.007 | -0.033 | 0.048 | 0.717 | n.s. |
| **Awake vs Moderate Propofol** | -0.017 | -0.037 | 0.003 | 0.099 | . |
| **Mild vs Moderate Propofol** | -0.010 | -0.030 | 0.010 | 0.333 | n.s. |
| **Recovery vs Moderate Propofol** | 0.011 | -0.010 | 0.032 | 0.302 | n.s. |
| **Awake vs fMRI+** | -0.003 | -0.052 | 0.045 | 0.898 | n.s. |
| **Awake vs Mild Propofol** | -0.007 | -0.025 | 0.011 | 0.454 | n.s. |
| **Scan 1 vs Scan 2** | -0.028 | -0.046 | -0.009 | 0.003 | ** |
| **Awake vs Recovery** | -0.001 | -0.015 | 0.013 | 0.837 | n.s. |
| **Placebo vs Ketamine** | -0.007 | -0.016 | 0.003 | 0.164 | n.s. |
| **Placebo vs LSD** | -0.014 | -0.023 | -0.005 | 0.002 | ** |

**Supplementary Table 3.** LME results for repertoire diversity of the connectome harmonics, from rotated harmonics. * $p < 0.05$; ** $p < 0.01$; *** $p < 0.001$; ** $p < 0.01$; . $p < 0.10$; n.s. not significant.

| Contrast | Fixed Effect | 95% CI Lower | 95% CI Upper | p-value | Significance |
|---|---|---|---|---|---|
| **Awake vs fMRI-** | -0.030 | -0.045 | -0.014 | 1.338E-04 | *** |
| **fMRI+ vs fMRI-** | -0.040 | -0.054 | -0.026 | 4.124E-08 | *** |
| **Awake vs DOC** | -0.014 | -0.027 | -0.001 | 0.039 | * |
| **Awake vs Moderate Propofol** | -0.018 | -0.034 | -0.003 | 0.023 | * |
| **Mild vs Moderate Propofol** | -0.017 | -0.033 | -0.001 | 0.038 | * |
| **Recovery vs Moderate Propofol** | -0.001 | -0.018 | 0.016 | 0.920 | n.s. |
| **Awake vs fMRI+** | 0.008 | -0.005 | 0.021 | 0.209 | n.s. |
| **Awake vs Mild Propofol** | -0.001 | -0.015 | 0.013 | 0.861 | n.s. |
| **Scan 1 vs Scan 2** | -0.017 | -0.032 | -0.002 | 0.023 | * |
| **Awake vs Recovery** | -0.003 | -0.012 | 0.006 | 0.494 | n.s. |
| **Placebo vs Ketamine** | 0.003 | -0.003 | 0.009 | 0.280 | n.s. |
| **Placebo vs LSD** | -0.003 | -0.010 | 0.003 | 0.334 | n.s. |

**Supplementary Table 4.** LME results for repertoire diversity of the connectome harmonics, from a randomised connectome. * $p < 0.05$; ** $p < 0.01$; *** $p < 0.001$; ** $p < 0.01$; . $p < 0.10$; n.s. not significant.

| Contrast | Fixed Effect | 95% CI Lower | 95% CI Upper | p-value | Significance |
|---|---|---|---|---|---|
| Awake vs fMRI- | -0.059 | -0.106 | -0.012 | 0.014 | * |
| fMRI+ vs fMRI- | -0.067 | -0.120 | -0.014 | 0.013 | * |
| Awake vs DOC | -0.028 | -0.071 | 0.015 | 0.198 | n.s. |
| Awake vs Moderate Propofol | -0.036 | -0.050 | -0.022 | 8.799E-07 | *** |
| Mild vs Moderate Propofol | -0.039 | -0.054 | -0.025 | 1.211E-07 | *** |
| Recovery vs Moderate Propofol | -0.034 | -0.050 | -0.018 | 1.997E-05 | *** |
| Awake vs fMRI+ | 0.010 | -0.035 | 0.055 | 0.671 | n.s. |
| Awake vs Mild Propofol | 0.003 | -0.009 | 0.016 | 0.604 | n.s. |
| Scan 1 vs Scan 2 | -0.002 | -0.016 | 0.012 | 0.794 | n.s. |
| Awake vs Recovery | 0.000 | -0.006 | 0.006 | 0.997 | n.s. |
| Placebo vs Ketamine | 0.015 | 0.011 | 0.020 | 3.149E-12 | *** |
| Placebo vs LSD | 0.039 | 0.033 | 0.044 | p<E-10 | *** |

**Supplementary Table 5.** LME results for repertoire diversity of the connectome harmonics, obtained from the HCP-985 connectome. * $p < 0.05$; ** $p < 0.01$; *** $p < 0.001$; ** $p < 0.01$; . $p < 0.10$; n.s. not significant.

| Contrast | Fixed Effect | 95% CI Lower | 95% CI Upper | p-value | Significance |
|---|---|---|---|---|---|
| **Awake vs fMRI-** | -0.059 | -0.108 | -0.009 | 0.021 | * |
| **fMRI+ vs fMRI-** | -0.068 | -0.123 | -0.012 | 0.017 | * |
| **Awake vs DOC** | -0.027 | -0.072 | 0.018 | 0.232 | n.s. |
| **Awake vs Moderate Propofol** | -0.037 | -0.051 | -0.023 | 4.977E-07 | *** |
| **Mild vs Moderate Propofol** | -0.039 | -0.054 | -0.025 | 1.421E-07 | *** |
| **Recovery vs Moderate Propofol** | -0.034 | -0.050 | -0.018 | 2.379E-05 | *** |
| **Awake vs fMRI+** | 0.011 | -0.037 | 0.059 | 0.655 | n.s. |
| **Awake vs Mild Propofol** | 0.002 | -0.010 | 0.015 | 0.711 | n.s. |
| **Scan 1 vs Scan 2** | -0.003 | -0.017 | 0.011 | 0.680 | n.s. |
| **Awake vs Recovery** | 0.001 | -0.005 | 0.007 | 0.736 | n.s. |
| **Placebo vs Ketamine** | 0.015 | 0.011 | 0.020 | 1.653E-12 | *** |
| **Placebo vs LSD** | 0.043 | 0.038 | 0.049 | p<E-10 | *** |

**Supplementary Table 6.** LME results for repertoire diversity of the connectome harmonics, obtained from the MGH-32 connectome. * $p < 0.05$; ** $p < 0.01$; *** $p < 0.001$; . $p < 0.10$; n.s. not significant.

# Supplementary References


1. Atasoy, S., Roseman, L., Kaelen, M., Kringelbach, M. L., Deco, G. & Carhart-Harris, R. L. Connectome-harmonic decomposition of human brain activity reveals dynamical repertoire re-organization under LSD. *Scientific Reports* **7**, 1–18 (2017).
