# Supplementary material for: Distributed harmonic patterns of structure-function dependence orchestrate human consciousness
Source: Commun Biol. 2023 Jan 28;6:117. doi: 10.1038/s42003-023-04474-1 (PMC9884288; doi:10.1038/s42003-023-04474-1)
Supplement: Supplementary file 2 — Description of Additional Supplementary Data [file 42003_2023_4474_MOESM2_ESM.docx]

**Description of Additional Supplementary Files**

**File name:** Supplementary Data 1

**Description:** The source data behind the graphs in Figure 3.

**File name:** Supplementary Data 2

**Description:** The source data behind the graphs in Figure 4.

**File name:** Supplementary Data 3

**Description:** The source data behind the graphs in Figure 5.
