# Supplementary material for: Distributed harmonic patterns of structure-function dependence orchestrate human consciousness
Source: Commun Biol. 2023 Jan 28;6:117. doi: 10.1038/s42003-023-04474-1 (PMC9884288; doi:10.1038/s42003-023-04474-1)
Supplement: Supplementary file 6 — Reporting summary [file 42003_2023_4474_MOESM6_ESM.pdf]

nature research

Corresponding author(s): Andrea I. Luppi (al857@cam.ac.uk)

Last updated by author(s): Dec 30, 2022

Reporting Summary

Nature Research wishes to improve the reproducibility of the work that we publish. This form provides structure for consistency and transparency in reporting. For further information on Nature Research policies, see our Editorial Policies and the Editorial Policy Checklist.

Statistics

For all statistical analyses, confirm that the following items are present in the figure legend, table legend, main text, or Methods section.

n/a	Confirmed	
☐	☒	The exact sample size (*n*) for each experimental group/condition, given as a discrete number and unit of measurement
☐	☒	A statement on whether measurements were taken from distinct samples or whether the same sample was measured repeatedly
☐	☒	The statistical test(s) used AND whether they are one- or two-sided<br>*Only common tests should be described solely by name; describe more complex techniques in the Methods section.*
☐	☒	A description of all covariates tested
☐	☒	A description of any assumptions or corrections, such as tests of normality and adjustment for multiple comparisons
☐	☒	A full description of the statistical parameters including central tendency (e.g. means) or other basic estimates (e.g. regression coefficient) AND variation (e.g. standard deviation) or associated estimates of uncertainty (e.g. confidence intervals)
☐	☒	For null hypothesis testing, the test statistic (e.g. F, t, r) with confidence intervals, effect sizes, degrees of freedom and P value noted<br>*Give P values as exact values whenever suitable.*
☒	☐	For Bayesian analysis, information on the choice of priors and Markov chain Monte Carlo settings
☒	☐	For hierarchical and complex designs, identification of the appropriate level for tests and full reporting of outcomes
☐	☒	Estimates of effect sizes (e.g. Cohen's d, Pearson's r), indicating how they were calculated

Our web collection on statistics for biologists contains articles on many of the points above.

Software and code

Policy information about availability of computer code

Data collection	A computerized pump (Graseby 3500, Graseby Medical, UK) was used for ketamine infusion, and an Alaris PK infusion pump (Carefusion, Basingstoke, UK) for propofol infusion.
Data analysis	The Java Information Dynamics Toolbox, together with together with Python and Octave/Matlab interfaces, has been made freely available online: https://github.com/jlizier/jidt. Code for the spherical rotations has been made freely available online: github.com/spin-test/spin-test. The CONN toolbox v17f is freely available online (http://www.nitrc.org/projects/conn). DSI Studio is freely available online: http://dsi-studio.labsolver.org. Lead-DBS is freely available online: http://www.lead-dbs.org. The Brain Connectivity Toolbox is freely available online: https://sites.google.com/site/bctnet/.

For manuscripts utilizing custom algorithms or software that are central to the research but not yet described in published literature, software must be made available to editors and reviewers. We strongly encourage code deposition in a community repository (e.g. GitHub). See the Nature Research guidelines for submitting code & software for further information.

Data

Policy information about availability of data

All manuscripts must include a data availability statement. This statement should provide the following information, where applicable:

- Accession codes, unique identifiers, or web links for publicly available datasets
- A list of figures that have associated raw data
- A description of any restrictions on data availability

The datasets analysed during the current study can be made available on request from the following authors. Propofol anaesthesia, test-retest and Disorders of Consciousness datasets: Dr. Emmanuel A. Stamatakis (University of Cambridge, Division of Anaesthesia; email: eas46@cam.ac.uk). LSD dataset: Dr. Robin L. Carhart-Harris (Imperial College London, Centre for Psychedelic Research; email: r.carhart-harris@imperial.ac.uk). Ketamine dataset: Dr. Ram Adapa (University of

Cambridge, Division of Anaesthesia; email: ra342@cam.ac.uk). The Human Connectome Project datasets are freely available from http://www.humanconnectome.org/.

Field-specific reporting

Please select the one below that is the best fit for your research. If you are not sure, read the appropriate sections before making your selection.

☐ Life sciences ☒ Behavioural & social sciences ☐ Ecological, evolutionary & environmental sciences

For a reference copy of the document with all sections, see nature.com/documents/nr-reporting-summary-flat.pdf

Behavioural & social sciences study design

All studies must disclose on these points even when the disclosure is negative.

Study description	This study uses quantitative methods.
Research sample	Ketamine dataset: 21 participants (10 males; mean age 28.7 years, SD = 3.2 years); 20 were included in the present study. Propofol dataset: 25 healthy volunteers (11 males, 14 females; mean age 34.7 years, SD = 9.0 years). 15 were included in the present study (7 males, 8 females). DOC patients: 71 patients meeting diagnostic criteria for Unresponsive Wakefulness Syndrome/Vegetative State or Minimally Conscious State were initially recruited for the study, and the final cohort analysed in this study comprised 22 individuals (14 males; 17 -70 years; mean time post injury: 13 months; 10 UWS, 12 MCS). LSD dataset: 20 healthy volunteers; 15 are included in the present analysis (11 males, 4 females; mean age 30.5 years, SD = 8.0 years) . Test-retest dataset: 22 healthy volunteers (age range, 19–57 years; mean age, 35.0 years; SD 11.2; female-to-male ratio, 9/13); 18 are included in the present analysis.
Sampling strategy	No power analysis was performed prior to data collection; the final sample sizes are within the range reported in the literature.
Data collection	Ketamine dataset: The infusion was performed and monitored by a trained anesthetist (RA) who was unblinded for safety reasons, but who otherwise had minimal contact with participants. At all other times, participants were supervised by investigators blinded to the infusion protocol. Bilateral intravenous catheters were inserted into volunteers' forearms, one for infusion, and the other for serial blood sampling. Infusion was performed using a computerized pump (Graseby 3500, Graseby Medical, UK). Blood samples were drawn before and after the resting fMRI scan and then placed on ice. Plasma was obtained by centrifugation and stored at −70° C. Plasma ketamine concentrations were measured by gas chromatography–mass spectrometry.<br>Propofol dataset: Two senior anaesthetists were present during scanning sessions and observed the subjects throughout the study from the MRI control room and on a video link that showed the subject in the scanner. They could not be blinded to experimental condition, since part of their role involved determining the participants' level of anaesthesia. Electrocardiography and pulse oximetry were performed continuously, and measurements of heart rate, non-invasive blood pressure, and oxygen saturation were recorded at regular intervals. Propofol was administered intravenously as a "target controlled infusion" (plasma concentration mode), using an Alaris PK infusion pump (Carefusion, Basingstoke, UK). Blood samples were drawn towards the end of each titration period and before the plasma target was altered, to assess plasma propofol levels.<br>LSD dataset: participants were blind to the experimental condition (LSD vs placebo) but the researchers were not. For infusion, a cannula was inserted into a vein in the antecubital fossa by a medical doctor and secured. All participants received 75 µg of LSD, administered intravenously via a 10ml solution infused over a two minute period, followed by an infusion of saline.<br>DOC dataset: in addition to the researcher and radiographer, a research nurse was also present. Since the patients' status as DOC patients was evident, no researcher blinding was possible.<br>Test-retest dataset: no experimental manipulation was present for the purposes of the present resting-state analysis.
Timing	Ketamine dataset: collected between March and July 2010;<br>Propofol dataset: the first 16 volunteers were scanned between June and October 2008; additional 9 volunteers were scanned between March 2009 and November 2011;<br>DOC patient data were collected between January 2010 and December 2015;<br>LSD dataset: data were collected between July 2014 and September 2015.<br>Test-retest dataset: data were collected between October 2009 and September 2010.
Data exclusions	Ketamine dataset: one participant was excluded due to excessive movement.<br>Propofol dataset: 10 participants were excluded, either because of missing scans (n=2), or due of excessive motion in the scanner (n=8, 5mm maximum motion threshold).<br>DOC dataset: Out of 71 DOC patients recruited for the study, individuals were systematically excluded from the final cohort analysed in this study based on the following criteria: 1) large focal brain damage (i.e. more than 1/3 of one hemisphere) as stated by an expert in neuroanatomy blinded to the patients' diagnoses; 2) excessive head motion during resting state scaning (i.e. greater than 3mm in translation and/or 3 degrees in rotation); 3) suboptimal segmentation and normalization of images. Thus, the final cohort analysed in this study comprised 22 patients.<br>LSD dataset: four participants were excluded for excessive motion (defined as 15% of volumes with mean frame-wise displacement > 0.5).<br>Test-retest dataset: participants could only be included if they had usable data for both scanning sessions. This left N=18 participants.<br>All exclusions took place before the data were analysed.
Non-participation	One participant from the LSD study aborted the experiment due to anxiety concerns.
Randomization	Ketamine dataset: this was a within-subjects design, and the order of administration (ketamine vs placebo) was randomised.<br>Propofol dataset: no randomisation was present: all participants were run in all conditions (awake, mild, moderate and recovery)

since this was a within-subjects design.
Test-retest dataset: these were healthy volunteers rectuited to act as controls for a patient group.
DOC dataset: patients were drawn from the local patient population; stratification into groups was not random, because it was based on fMRI task performance.
LSD dataset: this was a within-subjects design, and the order of administration LSD vs placebo) was randomised.

Reporting for specific materials, systems and methods

We require information from authors about some types of materials, experimental systems and methods used in many studies. Here, indicate whether each material, system or method listed is relevant to your study. If you are not sure if a list item applies to your research, read the appropriate section before selecting a response.

Materials & experimental systems

n/a	Involved in the study
☒	☐ Antibodies
☒	☐ Eukaryotic cell lines
☒	☐ Palaeontology and archaeology
☒	☐ Animals and other organisms
☐	☒ Human research participants
☒	☐ Clinical data
☒	☐ Dual use research of concern

Methods

n/a	Involved in the study
☒	☐ ChIP-seq
☒	☐ Flow cytometry
☐	☒ MRI-based neuroimaging

Human research participants

Policy information about studies involving human research participants

Population characteristics

See above.

Recruitment

Ketamine dataset: Volunteers were recruited via advertisements placed throughout central Cambridge, UK, and provided written informed consent. All participants underwent a screening interview in which they were asked whether they had previously been diagnosed or treated for any mental health problems and whether they had ever taken any psychotropic medications. Participants reporting a personal history of any mental health problems or a history of any treatment were excluded from the study. All participants were right-handed, were free of current of previous psychiatric or neurological disorder or substance abuse problems, and had no history of cardiovascular illness or family history of psychiatric disorder/ substance abuse.
Propofol dataset: healthy volunteers were recruited via advertisements placed throughout central Cambridge, UK. Exclusion criteria included history of psychiatric or neurological disorders, or contraindications to MRI scanning. All participants provided written informed consent.
Disorders of consciousness patients: Patients were referred from specialist rehabilitation settings as well as specialist nursing homes specifically identified in the ethics. All patients had been seen by a consultant neurologist or rehabilitation consultant before referral, to make diagnosis as part of multidisciplinary assessments. To be included in the study, patients must have had a DOC diagnosis, written informed consent to participation from their legal representative as well as the consent of their treating physician, and they must be capable of being transported to Addenbrooke's Hospital. The exclusion criteria included any medical condition that made it unsafe for the patient to participate (decision made by clinical personnel blinded to the specific aims of the study) or any reason they are unsuitable to enter the MRI scanner environment (e.g. non-MRI-safe implants), significant pre-existing mental health problems, or insufficient English pre injury.
LSD dataset: All participants were recruited via word of mouth and provided written informed consent to participate after study briefing and screening for physical and mental health. The screening for physical health included electrocardiogram (ECG), routine blood tests, and urine test for recent drug use and pregnancy. A psychiatric interview was conducted and participants provided full disclosure of their drug use history. Key exclusion criteria included: < 21 years of age, personal history of diagnosed psychiatric illness, immediate family history of a psychotic disorder, an absence of previous experience with a classic psychedelic drug (e.g. LSD, mescaline, psilocybin/magic mushrooms or DMT/ayahuasca), any psychedelic drug use within 6 weeks of the first scanning day, pregnancy, problematic alcohol use (i.e. > 40 units consumed per week), or a medically significant condition rendering the volunteer unsuitable for the study.
Test-retest dataset: Right-handed healthy participants were recruited via advertisements in the Cambridge area and were paid for their participation. Cambridgeshire 2 Research Ethics Committee approved the study (LREC 08/H0308/246) and all volunteers gave written informed consent before participating. Exclusion criteria included National Adult Reading Test (NART) <70, Mini Mental State Examination (MMSE) <23, left- handedness, history of drug/alcohol abuse, history of psychiatric or neurological disorders, contraindications for MRI scanning, medication that may affect cognitive performance or prescribed for depression, and any physical handicap that could prevent the completion of testing.

Ethics oversight

Ketamine dataset: Cambridge Local Research and Ethics Committee.
Propofol dataset: Cambridgeshire 2 Regional Ethics Committee.
LSD dataset: This study was approved by the National Research Ethics Service Committee London–West London and was conducted in accordance with the revised declaration of Helsinki (2000), the International Committee on Harmonization Good Clinical Practice guidelines and National Health Service Research Governance Framework. Imperial College London sponsored the research, which was conducted under a Home Office license for research with schedule 1 drugs.
DOC dataset: Ethical approval for testing patients was provided by the National Research Ethics Service (National Health

Service, UK; LREC reference 99/391).
Test-retest dataset: Cambridgeshire 2 Research Ethics Committee approved the study (LREC 08/H0308/246) .

Note that full information on the approval of the study protocol must also be provided in the manuscript.

Magnetic resonance imaging

Experimental design

Design type	Resting-state
Design specifications	Ketamine dataset: 10 minutes.<br>Propofol dataset: 5 minutes for each condition (awake, mild, moderate, recovery).<br>DOC dataset: 10 minutes.<br>LSD dataset: The precise length of each of the two BOLD scans included here was 7:20 minutes.<br>Test-retest dataset: 5:20 minutes.
Behavioral performance measures	For both DOC and propofol datasets, lack of behavioural responsiveness was used as an indicator of unconsciousness. For the DOC patients, it was assessed clinically at multiple times over the period of their stay in Addenbrooke's Hospital (Cambridge, UK). For the propofol dataset, the level of sedation in terms of behavioural responsiveness was assessed verbally immediately before and after each of the scanning runs. For the LSD dataset, Visual Analog Scale intensity ratings were collected after each scan, phrased as follows: "Please rate the intensity of the drug effects during the last scan", with a bottom anchor of "no effects", a mid-point anchor of "moderately intense effects" and a top anchor of "extremely intense effects".

Acquisition

Imaging type(s)	Functional and anatomical.
Field strength	3 Tesla (all datasets).
Sequence & imaging parameters	Ketamine dataset: Imaging parameters were: 3x3x3.75mm voxel size, with a time-to-repetition (TR) of 2000 ms, time-to-echo (TE) of 30 ms, flip angle of 781 in 64x64 matrix size, and 240mm field of view (FOV). A total of 300 volumes comprising 32 slices each were obtained. In addition, high- resolution anatomical T1 images were acquired using a three-dimensional magnetic-prepared rapid gradient echo (MPPRAGE) sequence. In all, 176 contiguous sagittal slices of 1.0mm thickness using a TR of 2300 ms, TE of 2.98 ms, flip angle of 91, and a FOV of 256mm in 240x256 matrix were acquired with a voxel size of 1.0mm3.<br>Propofol dataset: Each functional BOLD volume consisted of 32 interleaved, descending, oblique axial slices, 3 mm thick with interslice gap of 0.75 mm and in-plane resolution of 3 mm, field of view = 192x192 mm, repetition time = 2000 ms, acquisition time = 2 s, time echo = 30 ms, and flip angle 78. We also acquired T1-weighted structural images at 1 mm isotropic resolution in the sagittal plane, using an MPRAGE sequence with TR = 2250 ms, TI = 900 ms, TE = 2.99 ms and flip angle = 9 degrees, for localization purposes.<br>DOC dataset: Functional images (32 slices) were acquired using an echo planar sequence, with the following parameters: 3 x 3 x 3.75mm resolution, TR = 2000ms, TE = 30ms, 78 degrees FA. Anatomical scanning was also performed, acquiring high-resolution T1-weighted images with an MPRAGE sequence, using the following parameters: TR = 2300ms, TE = 2.47ms, 150 slices, resolution 1 x 1 x 1mm.<br>LSD dataset: BOLD-weighted fMRI data were acquired using a gradient echo planer imaging sequence, TR/TE = 2000/35ms, FoV = 220mm, 64x64 acquisition matrix, parallel acceleration factor = 2, 90 flip angle. Thirty five oblique axial slices were acquired in an interleaved fashion, each 3.4mm thick with zero slice gap (3.4mm isotropic voxels). High-resolution anatomical images were acquired with 3D fast spoiled gradient echo scans in an axial orientation, with field of view = 256x256x192 and matrix = 256x256x129 to yield 1mm isotropic voxel resolution. TR/TE = 7.9/3.0ms; inversion time = 450ms; flip angle = 20.<br>Test-retest dataset: resting-state fMRI was acquired for 5:20 minutes using a Siemens Trio 3T scanner (Erlangen, Germany). Functional imaging data were acquired using an echoplanar imaging (EPI) sequence with parameters TR 2,000 ms, TE 30 ms, Flip Angle 78◦, FOV 192 × 192mm2, in-plane resolution 3.0 × 3.0mm, 32 slices 3.0mm thick with a gap of 0.75mm between slices. A 3D high resolution MPRAGE structural image was also acquired, with the following parameters: TR 2,300 ms, TE 2.98 ms, Flip Angle 9◦, FOV 256 × 256 mm2.
Area of acquisition	Whole-brain.

Diffusion MRI ☒ Used ☐ Not used

Parameters	The spatial resolution was 1.25 mm isotropic. TR=5500ms, TE=89.50ms. The b-values were 1000, 2000, and 3000 s/mm2. The total number of diffusion sampling directions was 90, 90, and 90 for each of the shells in addition to 6 b0 images.

Preprocessing

Preprocessing software	We used the CONN toolbox version 17f, based on SPM12. The default pipeline was used. The pipeline comprised the following steps: removal of the first 5 volumes to allow magnetisation to reach steady state; functional realignment and motion correction; slice-timing correction to account for differences in time of acquisition between slices; identification of outlier scans by means of the quality assurance/artifact rejection software ART; spatial normalisation to Montreal Neurological Institute (MNI-152) standard space with 2mm isotropic resampling resolution, using the segmented grey matter image from each volunteer's high-resolution T1-weighted image, together with an a priori grey matter template.<br>Due to the presence of deformations caused by brain injury, rather than relying on automated pipelines, DOC patients' brains

were individually preprocessed using SPM12, with visual inspections after each step, and subsequently denoised following the steps outlined above within the CONN toolbox.

Normalization	Direct normalisation to MNI space (nonlinear) using the segmented grey matter image from each volunteer's high-resolution T1-weighted image, together with an a priori grey matter template.
Normalization template	MNI152 template.
Noise and artifact removal	The anatomical CompCor method was used, regressing out of the functional data the following confounding effects: the first five principal components attributable to each individual's white matter signal, and the first five components attributable to individual cerebrospinal fluid (CSF) signal; six subject-specific realignment parameters (three translations and three rotations) as well as their first- order temporal derivatives; the nuisance regressors identified by ART; and main effect of scanning condition. Linear detrending was also applied, and the subject-specific denoised BOLD signal timeseries were band-pass filtered to eliminate both low-frequency drift effects and high-frequency noise, thus retaining frequencies between 0.008 and 0.09 Hz. For the DOC patients, data underwent additional despiking with a hyperbolic tangent squashing function.
Volume censoring	The artifact rejection tool (ART), implemented in the CONN toolbox, was used to identify and regress out outlying volumes, as part of the CompCor denoising procedure described above. The default CONN settings of 5 global signal z-values and 0.9mm were used.<br>For the ketamine dataset, the mean percentage of volumes identified as artifacts was 0.9% (placebo) and 2.4% (ketamine).<br>For the propofol dataset, the mean percentage of volumes identified as artifacts was 2.0% (awake), 2.2% (mild sedation), 4.6% (moderate anaesthesia), and 6.2% (recovery).<br>For the DOC patients, the mean percentage of volumes identified as artifacts was 10.6%.<br>For the LSD dataset, the mean percentage of volumes identified as artifacts was 1.2% (placebo) and 1.6% (LSD).

Statistical modeling & inference

Model type and settings	Linear Mixed Effects models (implemented as the MATLAB function fitlme) were used to assess the statistical significance of the differences between conditions (states of consciousness).
Effect(s) tested	We treated condition (state of consciousness: for example, placebo vs LSD) as a fixed effect, and subjects as random effects. When one measurement was obtained for each timepoint, timepoints were also included as random effects, nested within subjects. For between-groups comparisons, age and biological sex were included as covariates of no interest.

Specify type of analysis: ☒ Whole brain ☐ ROI-based ☐ Both

Statistic type for inference<br>(See Eklund et al. 2016)	The connectome harmonics obtained from the whole brain were grouped into 15 logarithmically-spaced, frequency-specific bins; analyses were performed either separately for each harmonic bin (mass univariate approach, with False Discovery Rate correction for multiple comparisons).
Correction	The False Discovery Rate across 15 frequency bins (or 14 in the replication with different connectomes, and 25 in the replication with a different number of harmonic bins) was controlled by means of the Benjamini-Hochberg procedure.

Models & analysis

n/a	Involved in the study
☒	☐ Functional and/or effective connectivity
☒	☐ Graph analysis
☐	☒ Multivariate modeling or predictive analysis

Multivariate modeling and predictive analysis	Partial least squares (PLS, also known as Projection on Latent Spaces) is a multivariate technique used to identify relationships between sets of variables X (predictors) and Y (targets), in order to extract principal components as linear combinations of variables in each set that maximally covary with each other. In the present case, for each pair of states of consciousness under comparison, X was the matrix of binned energy values per subject (after averaging over timepoints) , and Y was the vector of binary classification between the two states (here, target vs baseline state of consciousness, e.g. anaesthetised vs awake, ketamine vs placebo, fMRI- vs fMRI+ DOC, etc) – making this an application of Partial Least Squares Discriminant Analysis (PLS-DA), owing to the binary nature of Y.
